# Amelioration of Metabolic Syndrome by Co-Administration of *Lactobacillus johnsonii* CRL1231 and Wheat Bran in Mice via Gut Microbiota and Metabolites Modulation

**DOI:** 10.3390/metabo15070466

**Published:** 2025-07-09

**Authors:** Matias Russo, Antonela Marquez, Estefanía Andrada, Sebastián Torres, Arlette Santacruz, Roxana Medina, Paola Gauffin-Cano

**Affiliations:** 1 Centro de Referencia para Lactobacilos (CERELA-FML-FECIC-CONICET), San Miguel de Tucumán T4000ILC, Tucumán, Argentina; mrusso@cerela.org.ar (M.R.); amarquez@cerela.org.ar (A.M.); eandrada@cerela.org.ar (E.A.); 2 Facultad de Ciencias de la Salud, Universidad del Norte Santo Tomas de Aquino, San Miguel de Tucumán T4000ILC, Tucumán, Argentina; 3 Facultad de Agronomía y Zootecnia, Universidad Nacional de Tucumán, San Miguel de Tucumán T4000ILC, Tucumán, Argentina; 4 Instituto de Bioprospección y Fisiología Vegetal (INBIOFIV-CONICET-UNT), San Miguel de Tucumán T4000ILC, Tucumán, Argentina; storres@csnat.unt.edu.ar; 5 Facultad de Ciencias Naturales e IML, Universidad Nacional de Tucumán (UNT), San Miguel de Tucumán T4000ILC, Tucumán, Argentina; 6 School of Engineering and Science, Tecnologico de Monterrey, Av. Eugenio Garza Sada 2501 Sur, Monterrey C.P. 64849, Nuevo León, Mexico; asantacruz@tec.mx

**Keywords:** metabolic syndrome, intestinal microbiota, probiotic, *Lactobacillus johnsonii*, high fat diet, feruloyl esterase, ferulic acid

## Abstract

Background/Objectives: *Lactobacillus johnsonii* CRL1231 (*Lj* CRL1231) is a strain with feruloyl esterase (FE) activity that enhances ferulic acid (FA) release from wheat bran (WB) and has potential as a probiotic for metabolic syndrome (MS). Given the potential health benefits of FA and its microbial metabolites, this study aimed to evaluate the therapeutic effect of *Lj* CRL1231 co-administered with WB in a mouse model of metabolic syndrome (MS) induced by a high-fat diet (HFD). Methods: Mice were divided into three groups and fed for 14 weeks as follows: the Control group (standard diet), the MS group (HFD+WB), and the MS+Lj group (HFD+WB and *Lj* CRL1231-dose 10^8^ cells/day). Specifically, we analyzed the changes in the intestinal microbiota (IM), colonic FE activity, generation of FA-derived and fermentation metabolites, and metabolic and inflammatory parameters. Results: Improvements in the MS+Lj group compared to the MS group included the following: a—a 38% increase in colonic FE activity, leading to elevated levels of FA-derived metabolites (e.g., dihydroferulic, dihydroxyphenylpropionic, and hydroxyphenylpropionic acids); b—a significant shift in the IM composition, with a 3.4-fold decrease in Firmicutes and a 2.9-fold increase in Bacteroidetes; c—a decrease in harmful bacteria (*Desulfovibrio*) by 93%, and beneficial bacteria like *Bifidobacterium* increased significantly (6.58 log cells/g); d—a 33% increase in total SCFAs; e—a 26% reduction in the adiposity index; f—a 12% increase in HDL cholesterol and a 19% reduction in triglycerides; g—normalized glucose and insulin resulting in a 2-fold lower HOMA-IR index; h—an improved inflammatory profile by decreasing TNF-α, IFN-γ, and IL-6 (3-, 5-, and 2-fold, respectively) and increasing IL-10 by 2-fold; i—alleviation of liver damage by normalizing of transaminases AST (19.70 ± 2.97 U/L) and ALT (13.12 ± 0.88 U/L); j—evidence of reduced oxidative damage. Conclusions: The co-administration of *L. johnsonii* CRL1231 and WB exerts a synergistic effect in mitigating the features of MS in HFD-fed mice. This effect is mediated by modulation of the gut microbiota, increased release of bioactive FA-derived compounds, and restoration of metabolic and inflammatory homeostasis. This strategy represents a promising dietary approach for MS management through targeted microbiota–metabolite interactions.

## 1. Introduction

Metabolic syndrome (MS) has been the leading cause of morbidity and mortality in the world in recent years [1]. MS is a cluster of cardiometabolic risk factors and comorbidities, conveying a high risk of both cardiovascular disease (CVD) and type 2 diabetes (T2D) [2]. The International Diabetes Federation defines metabolic syndrome (MS) as central obesity plus at least two of the following components: increased triglyceride levels, reduced high-density lipoprotein cholesterol (HDLc), elevated blood pressure (hypertension), and elevated fasting plasma glucose (or prediabetes) [3]. MS has been associated with many other clinical conditions, including oxidative stress, pro-inflammatory state, hepatic steatosis, non-alcoholic fatty liver disease (NAFLD), prothrombotic state, cholesterol gallstone disease, and reproductive disorders [4].

The two basic forces spreading MS are the increase in high-fat fat-low fiber fast food consumption and the decrease in physical activity (imbalance of calorie intake and energy expenditure). However, the genetic/epigenetic makeup of individuals may also influence, like other factors, such as the quality and composition of food and the intestinal microbiota (IM) composition [5,6]. A high-fat diet (HFD) alters the IM, and it leads to an imbalance: a reduction in beneficial bacteria (lactic and butyric producers) and an increase in pro-inflammatory/pathogenic bacteria, which is directly related to the appearance of weight gain, accumulation of abdominal fat, chronic inflammation, and metabolism disorders [7,8]. Several studies have shown that metabolic stressors, including HFD, promote obesity, insulin resistance, and MS. But diet and lifestyle modifications may effectively prevent MS development, even more than pharmacological agents [9]. The problem with pharmaceutical preparations used in the treatment of MS is that they cause unwanted side effects and adverse reactions, such as gastrointestinal discomfort and liver damage [10]. Given this difficulty, probiotic dietary interventions have become a promising treatment option for MS due to their safety and effectiveness, mainly bacteria of the *Lactobacillus* genus [7,8]. Furthermore, several *Lactobacillus* (*L*.) strains can improve MS by modulating the IM and/or its metabolites [8].

*L. johnsonii* is often isolated from human and animal intestines [11]. Several probiotic characteristics have been reported in *L. johnsonii* strains, including antagonistic activity against intestinal pathogens, alleviation of T2D and dyslipidemias, or stimulation of the immune system [12]. *L. johnsonii* CRL1231 (*Lj* CRL1231) is a feruloyl esterase (FE)-producing strain capable of releasing ferulic acid (FA) from plant sources. This property can potentially improve the biomarkers involved in obesity by increasing the intestinal FE activity in mice [13]. However, the impact of *Lj* CRL1231 on the IM and the production of dietary fiber-derived metabolites with possible implications for improving the MS have not yet been investigated.

Ferulic acid (FA) (4-hydroxy-3-methoxycinnamic acid) is a phenolic compound present in daily human food (coffee, fruits, cereals, and vegetables), and it exerts beneficial effects in improving glucose and lipid metabolism disorders [14]. FA possesses antioxidant, anti-inflammatory, antifibrosis, and anticancerogenic properties [15]. Notably, FA exerts protective effects on T2D and its associated complications, and it has also been used in the treatment of lung diseases, CVD, and cancer [14,16]. FA represents up to 90% of the phenolic acids present in whole wheat, mainly in the grain’s outer layers (the bran), where it is found chiefly in insoluble conjugated bound forms [17,18]. One of the principal food sources of FA is wheat bran (WB) [19]. WB fiber primarily contains FA bound to arabinoxylans in the cell wall through ester linkages, thus restricting its bioavailability and potential health benefits [18,20].

Therefore, the present study aimed to assess the nutritional, metabolic, oxidative, and inflammatory profiles of mice with MS following oral co-administration of *Lj* CRL1231 and WB dietary fiber. Additionally, this study evaluated the impact of this probiotic strain on the composition of the IM and the generation of health-promoting metabolites.

## 2. Materials and Methods

### 2.1. The Bacterial Strain Preparation

The strain *Lj* CRL1231 was obtained from the CERELA Culture Collection (CERELA FML-FECIC-CONICET, Tucumán, Argentina). This bacterial strain has FE activity and can release the FA from WB fibers [13]. *Lj* CRL1231 was stored at −80 °C in De Man–Rogosa–Sharpe (MRS) broth (Britania, Buenos Aires, Argentina) containing 20% (*v/v*) glycerol. For activation, the strain was inoculated in the MRS broth (2%, *v*/*v*), incubated at 37 °C for 18 h, and sub-cultured twice before each experiment. Cells were harvested by centrifugation at 10,000× *g* for 10 min and washed twice with sterile phosphate-buffered saline (PBS) pH 7.0. For the animal assays, the bacteria were collected by centrifugation (10,000× *g* for 10 min), washed twice with sterile phosphate-buffered saline (PBS) at pH 7.0, and then resuspended in sterile drinking water to a final concentration of 10^9^ CFU/mL. Bacteria were freshly prepared daily during the 14-week experiment.

The in vitro inhibitory capacity of α-glucosidase activity by *Lj* CRL1231 was determined using *p*-nitrophenyl α-D-glucopyranoside as a substrate, according to the method described by Li et al. (2016) [20].

### 2.2. Experimental Design: Animals and Diet

Male six-week-old Swiss albino mice were obtained from the closed colony at CERELA. The mice were housed in cages under environmentally controlled conditions (22 ± 2 °C with a 12-h light/12-h dark cycle). The Institutional Committee for the Care and Use of Laboratory Animals at CERELA approved this research under the protocol CRL-BIOT-EF-2019/1A.

After one week of acclimatization, the mice were randomly assigned to three groups (Supplementary Materials, Figure S1): the Control group, the MS group, and the MS+Lj group, with 8 mice in each group. These mice were fed daily for 14 weeks, as indicated in Table 1. The nutritional composition of the two diets (ND and HFD+WB) is specified in the Supplementary Materials, Table S1.

The animals’ food intake and body weight (BW) were monitored weekly during the experimental period. Body weight gain (BWG) and the food efficiency ratio (FER) were calculated at the end of week 14 according to the following equations:

$$BWG\;(g)=BW\;week\;14\;\left(g\right)-BW\;initial(g)$$

$$FER=\frac{BWG\;(g)}{Food\;consumed\;(g)}$$

### 2.3. Oral Glucose and Sucrose Tolerance Test

Oral glucose and sucrose tolerance tests were performed for all groups at week 14 after a 12-h fast. Fasting blood glucose levels were monitored in all mice using a commercial glucometer, Accu-Chek Active (Roche, Germany). A 2 g/kg glucose or sucrose solution was administered, and blood glucose levels were monitored every 30 min for 120 min [21].

### 2.4. Microbiota Characterization

#### 2.4.1. Bacterial DNA Extraction

At the end of the feeding period (14 weeks) and before sacrifice, half of the mice in each group (*n* = 4) were randomly chosen, and stool samples were collected, placed in sterile Eppendorf tubes, and stored at −20 °C. A commercial kit was used to extract DNA from the fecal samples (QIAamp DNA Stool Mini Kit, Hilden, Germany).

#### 2.4.2. Metagenomic Sequencing

Aliquots of 20 microliters of DNA extracted from each sample were used for amplicon sequencing with a MiSeq Illumina sequencing platform (Shallowater, TX, USA). The V4 region of the 16S rRNA gene was amplified by PCR, using a primer pair 515F (5′-GTGCCAGCMGCCGCGGTAA-3′) and 806R (5′-GGACTACHVGGGTWTCTAAT-3′) to generate an amplicon size of ∼400 bp. Adapters and sequencing indices were ligated to the purified PCR products using the Nextera XT Sample Preparation Kit (Illumina, San Diego, CA, USA) for library construction. Equimolar amounts of each library were then pooled and sequenced with the Illumina MiSeq personal sequencer. A QIIME pipeline was used to control the quality of the sequence [22]. First, quality filters were screened for chimeras using the UCHIME algorithm implemented in USEARCH (version 6.1544; 21) [23,24]. The remaining high-quality 16S rRNA gene sequences were clustered into OTUs at 97% similarity with the de novo reference OTU picking method and USEARCH (version 6.1544). A Bayesian RDP Classifier was used for taxonomy assignments with a confidence cutoff of 0.8. Alpha diversity metrics (Chao 1, Shannon, PD whole tree) were determined using the QIIME 2 release 2017.2 software, with sample rarefaction set at 18,850 sequences per sample based on the sample with the smallest number of sequences. Beta diversity metrics were also calculated using the QIIME 2 release 2017.2 software (employing weighted UniFrac distances and Bray–Curtis similarity) [23].

#### 2.4.3. Microbial Analysis by Quantitative PCR (qPCR)

Specific primers (Supplementary Materials, Table S2) targeting different bacterial genera and species were used to characterize the composition of microbiota by qPCR using LightCycler^®^ 480 SYBR Green I Master (Roche, Indianapolis, IN, USA) with an ABI PRISM 7000-PCR sequence detection system (Applied Biosystems, Warrington, UK), according to the methodology described by Cano et al. (2013) [25].

### 2.5. Animal Sacrifice and Sample Collection

At the end of week 14, all animals were sacrificed by injecting ketamine hydrochloride (100 mg per kg of body weight) and xylazine hydrochloride (5 mg per kg of body weight) into the peritoneal region. Blood samples were collected by cardiac puncture and were transferred into tubes containing EDTA (Wiener Lab, Rosario, Argentina).

Blood samples were centrifuged at 2500× *g* for 10 min at 4 °C to separate the plasma. The plasma was transferred to 1.5 mL Eppendorf tubes and stored at −20 °C until further analysis. All other internal organs, including adipose tissues (mesenteric and epididymal), large intestine, and liver, were immediately collected after animal sacrifice, washed with PBS pH 7.0, and weighed. Four liver and epididymal adipose tissue samples from each experimental group were stored in formaldehyde solution (10% *v*/*v* in PBS, pH 7.0) for histological analyses.

### 2.6. Biochemical Evaluations and Cardiovascular Risk Indices Calculations

Plasma concentrations of glucose, total cholesterol, HDL-cholesterol (HDLc), LDL-cholesterol (LDLc), and triglycerides (TG), and the activities of aspartate transaminase (AST), alanine transaminase (ALT) were measured by enzymatic methods using commercial kits (Wiener Lab, Rosario, Argentina).

Plasma leptin was determined using an ELISA immunoassay kit (DuoSet, R&D Systems, Minneapolis, MN, USA).

Plasma insulin was determined with a mouse insulin ELISA kit (ALPCO Diagnostics, Salem, NH, USA).

Furthermore, the homeostasis model assessment of basal insulin resistance (HOMA-IR) [21] and cardiovascular risk indicators (atherogenic index of plasma, Castelli risk index, and atherogenic coefficient) [21,26] were calculated at week 14 using the following formulas:

$$HOMA-IR\;index=\frac{Insuline\;\left(µU/mL\right)\times \;Glucose\;(mmol/L)\;}{22.5}$$

$$Atherogenic\;coefficient\;(AC)=\frac{Total\;cholesterol-HDLc}{HDLc}$$

$$Castelli’s\;risk\;index\;\left(CRI\right)\;\;\;\;\;\;\;CRI-I=\frac{Total\;cholesterol\;}{HDLc};\;CRI-II=\frac{LDLc\;}{HDLc}$$

$$Atherogenic\;index\;of\;plasma\;\left(AIP\right)=Log\;\frac{Triglycerides\;}{HDLc}$$

### 2.7. Cytokines Measurement

The concentration of cytokines was measured in plasma by cytometric bead array (CBA). Concentrations of interleukin-10 (IL-10), tumor necrosis factor (TNF-α), interferon-γ (IFN-γ), and IL-6 were determined using Mouse Flex Sets (BD Bioscience, San Diego, CA, USA) following the manufacturer’s instructions.

### 2.8. Plasma Lipoperoxidation

Lipid peroxides (malondialdehyde, MDA) were estimated in plasma according to Russo et al. (2020) [21]. The results were presented as the concentration (nmol/g protein) of thiobarbituric acid reactive substances (TBARS). The protein concentrations in the samples were determined using a commercial kit (Bio-Rad, Hercules, CA, USA).

### 2.9. Histopathological Analysis and Adiposity Index

Liver and epididymal adipose tissue (four from each group of mice) were taken for histological evaluation. Samples were fixed with formaldehyde solution (10% *v*/*v* in PBS, pH 7.0), dissected, and embedded in paraffin. Tissues were stained with hematoxylin–eosin (H&E) and examined under an optical microscope.

Carl Zeiss Axio Vision Release 4.8 Software was used to measure the adipocyte area. They were grouped by size ranges according to their areas (A) as follows: 500 ≤ A ≤ 1000 μm^2^; 1000 < A ≤ 2000 μm^2^; 2000 < A ≤ 3000 μm^2^; 3000 < A ≤ 4000 μm^2^; 4000 < A ≤ 8000 μm^2^.

The adiposity index was calculated using the following formula:

$$Adiposity\;index=\frac{Mesenteric\;fat\;\left(g\right)+Epididymal\;fat(g)}{Body\;weight\;(g)}\times 100$$

### 2.10. Assessment of Hepatic Oxidative Stress

Liver tissue (one gram) was homogenized in 5 mL of 100 mM potassium phosphate buffer (pH 7.5) containing 1 mM EDTA and 3 mM DL-dithiothreitol. The homogenate obtained was centrifuged (12,000× *g* for 30 min at 4 °C), and the supernatant was analyzed for antioxidant markers.

Glutathione peroxidase (GPx) and glutathione reductase (GR) activities were assayed using NADPH. Absorbance reduction at 340 nm was measured every minute for at least 5 min using a plate reader (Tecan Genios, A-5082, Grödig, Austria). One unit of GPx activity was defined as the amount of enzyme producing 1 nmol of oxidized NADP per minute. Similarly, one unit of GR activity was defined as the amount of enzyme producing 1 nmol of NADP per minute. In both cases, the results were presented as U/g liver [21].

### 2.11. Determination of Short-Chain Fatty Acids (SCFAs), FA Metabolites, and FE Esterase Activity in Large Intestine Contents

Large intestines from each group of mice were aseptically removed, and the intestinal contents were collected.

SCFAs were determined according to the methodology described by Russo et al. [21]. Acetic, propionic, and butyric acids present in the large intestine content were quantified by HPLC (Knauer system) with an ion-exchange column (Bio-Rad Aminex HPX-87H; 300 × 7.8 mm). The components were eluted with 5 mM H_2_SO_4_ at a flow rate of 0.6 mL/min.

The intestinal FE activity in the gut contents was determined according to Russo et al. [21]. One unit of FE activity was defined as the amount of enzyme capable of releasing 1 mmol of FA per hour. The results were expressed as specific activity for the units (U) of FE activity per gram of intestinal content.

The presence of the different phenolic compounds derived from FA metabolism (FA-derived metabolites) in intestinal contents was evaluated. For this, 30% (*w*/*v*) intestinal homogenates were prepared with PBS pH 7.0. An aliquot of 500 µL of homogenate was taken, and 10 µL of HCl (37%) was added (acidification), then 2 mL of ethyl acetate was added to extract the compounds of interest. It was centrifuged at 8000× *g* for 10 min, and the organic phase was recovered. Then, it was evaporated to dryness in a water bath at 40 °C under a stream of N_2_. The dry residue obtained after this process was resuspended in 100 µL of methanol. Finally, 20 µL of each sample was injected into an HPLC-UV-MS system (InMet S.A., Rosario, Argentina). The conditions were as follows: Mobile phase: A: H_2_O MQ/0.1% *v*/*v* formic acid; B: acetonitrile/0.1% formic acetic; flow: 0.2 mL/min; Column: C18 Hypersil-GOLD, Thermo Fisher Scientific, Waltham, MA, USA (50 × 2.1 mm; 1.9 μm particle size); column temperature: 25 °C; Autosampler temperature: 20 °C; Detection: UV 320 nm, 265 nm + MS; run time: 21 min.

### 2.12. Statistical Analysis

Statistical analyses were carried out using SPSS 12.0 software (SPSS Inc., Chicago, IL, USA). Data was normally distributed, and significant differences were determined by applying a one-way analysis of variance (ANOVA) with a post hoc Tukey’s test. In every case, *p*-values < 0.05 were considered statistically significant.

## 3. Results

### 3.1. Effect of Lj CRL1231 on Body Weight Gain (BWG), Food Efficiency Ratio (FER), Adiposity Index and Leptin Levels

**Figure 1:**
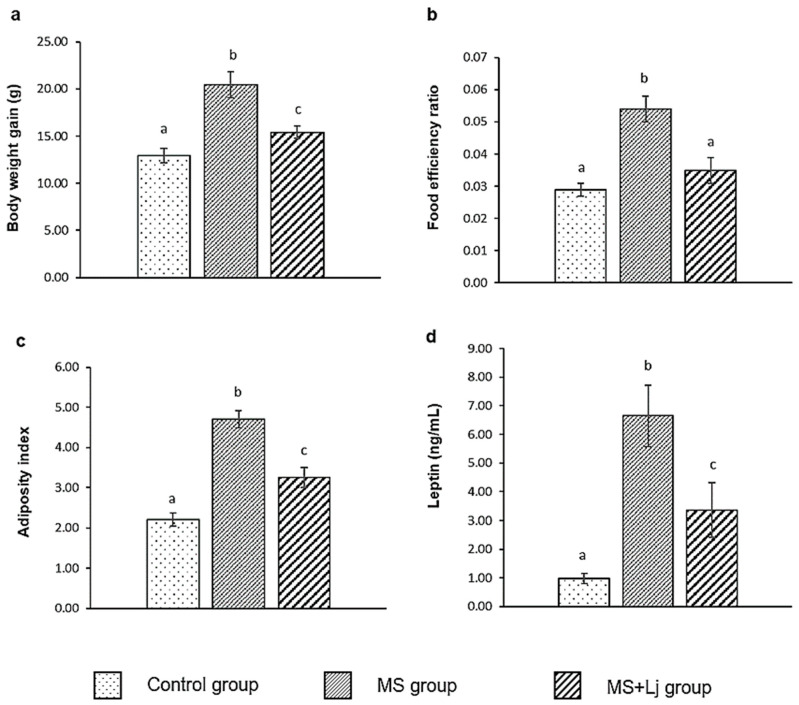
Evaluation of parameters related to body weight, food intake, and fat accumulation: (**a**) body weight gain (g); (**b**) food efficiency ratio; (**c**) adiposity index; (**d**) leptin levels (ng/mL). Data are expressed as mean ± SE (standard error), *n* = 8 per group. Values with different letters are significantly different (*p* < 0.05). Control: mice receiving normal diet; MS (Metabolic syndrome): mice receiving HFD+WB diet; MS+Lj: mice receiving HFD+WB diet and *L. johnsonii* CRL1231.

Consumption of HFD+WB for 14 weeks increased in BWG and FER by 62% and 85%, respectively, in the MS group compared to the Control group. Oral administration of *Lj* CRL1231 significantly reduced the BWG and FER in the MS+Lj group compared to the MS group, showing values closer to the Control group (Figure 1a,b).

Feeding mice with HFD+WB increased the weight of the mesenteric and epididymal adipose tissue; therefore, the adiposity index of the MS group was higher (98% increase) compared to the Control group. The MS+Lj mice that received probiotic supplementation accumulated less adipose tissue; a 26% decrease in the adiposity index compared to the MS group was observed (Figure 1c).

Plasma leptin levels were about 7 times higher in the MS group compared to the Control group. Leptin values in the MS+Lj group were halved (0.5-fold) compared to the MS group (Figure 1d).

### 3.2. Effect of Lj CRL1231 on Adipocyte Size

**Figure 2:**
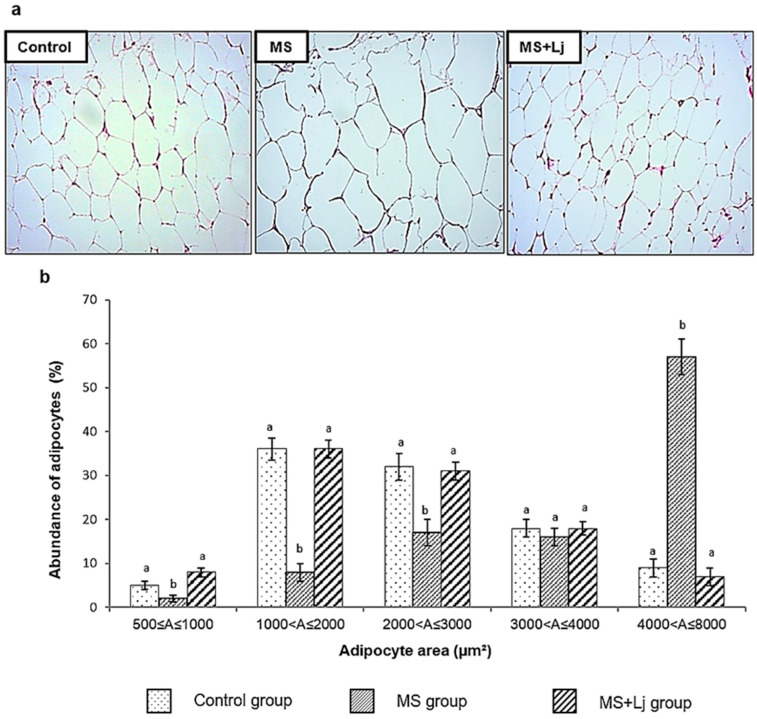
Effect of *L. johnsonii* CRL1231 on the adipocyte size of mice fed for 14 weeks. (**a**) Representative photomicrograph of epididymal adipose tissues. The histological sections were stained with hematoxylin–eosin, and the images were captured using a Carl ZEISS microscope with 20× magnification. (**b**) Abundance of adipocytes (%) according to their size (µm^2^). Data represent mean ± SE (standard error) of *n* = 8 mice per group. Values with different letters differ significantly in each area range (*p* < 0.05).

A histological analysis of the hematoxylin–eosin-stained sections revealed a change in the structure of the epididymal adipose tissue; when mice were fed with HFD+WB (MS group), they showed large adipocytes and, when in addition to this diet, they received *Lj* CRL1231 (MS+Lj group), the adipocyte architecture is like the Control group (Figure 2a).

Figure 2b shows that the MS group has a high abundance (58%) of larger adipocytes (4000 < A ≤ 8000 µm^2^), while in the Control and the MS+Lj groups, these large adipocytes represent only about 8% of the total amount. On the other hand, in the Control and the MS+Lj groups, there was a greater number of adipocytes of small size (1000 < A ≤ 2000 µm^2^) and medium size (2000 < A ≤ 3000 µm^2^), with an abundance of around 35% in each case; on the contrary, the MS group showed a low proportion of small and medium adipocytes (8% and 17%, respectively).

### 3.3. Effect of Lj CRL1231 on Inflammatory Profile

**Figure 3:**
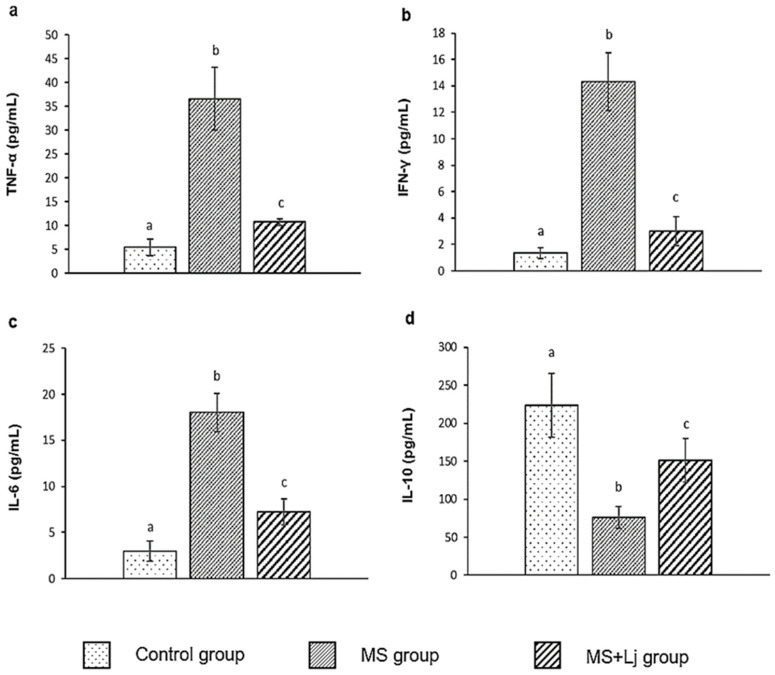
Evaluation of inflammatory status of mice by determination of cytokine levels: (**a**) TNF-α (pg/mL); (**b**) IFN-γ (pg/mL); (**c**) IL-6 (pg/mL); (**d**) IL-10 (pg/mL). Data are expressed as mean ± SE (standard error), *n* = 8 per group. Values with different letters are significantly different (*p* < 0.05). Control: mice receiving normal diet; MS (Metabolic syndrome): mice receiving HFD+WB diet; MS+Lj: mice receiving HFD+WB diet and *L. johnsonii* CRL1231.

Figure 3 shows the plasmatic levels of the cytokines TNF-α, IFN-γ, IL-6, and IL-10, determined by flow cytometry in all experimental groups. In the MS group, there was a significant increase in pro-inflammatory cytokines TNF-α, IFN-γ, and IL-6 (7-, 10-, and 6-fold increases, respectively) compared to the Control group. However, with the administration of *Lj* CRL1231, the levels of pro-inflammatory cytokines were reduced in the MS+Lj group compared to the MS group (3-, 5-, and 2-fold reduction in TNF-α, IFN-γ, and IL-6, respectively) (Figure 3a, b, and c). On the other hand, a 3-fold reduction in the anti-inflammatory cytokine IL-10 was observed in the MS group compared to the Control. In the MS+Lj group, values of IL-10 were 2-fold higher than in the MS group (Figure 3d).

### 3.4. Effect of Lj CRL1231 on Liver Injury

**Figure 4:**
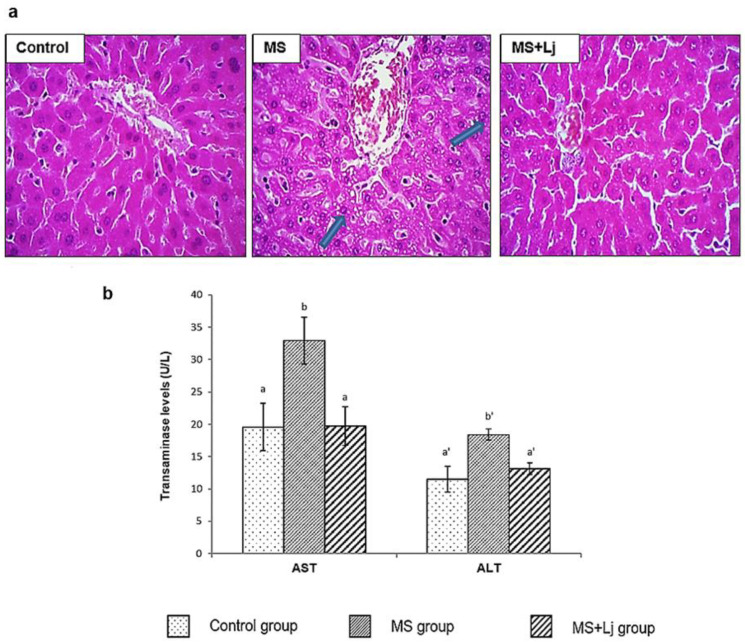
Effect of diet and administration of *L. johnsonii* CRL1231 in the liver of mice at week 14 of feeding. (**a**). Representative photomicrograph of the liver of mice. The histological sections were stained with hematoxylin–eosin, and the images were captured using a Carl ZEISS microscope with 200x magnification. The arrows indicate some alterations observed in the MS group (lipid drops and binucleation). (**b**). Aspartate aminotransferase (AST) and alanine aminotransferase (ALT) concentrations (U/L). Data represent mean ± SE (standard error) of *n* = 8 mice. Values with different letters differ significantly (*p* < 0.05).

Figure 4 presents histopathological images of liver sections from the three groups of mice at the end of 14 weeks of feeding. As a result of the consumption of HFD+WB, the MS group exhibited fatty infiltration as a manifestation of hepatic steatosis (presence of numerous lipid vacuoles (droplets) and cytoplasmic granularity in the hepatocyte cells); in addition, ballooning and hepatocellular binucleation were observed. Supplementation with *Lj* CRL1231 to mice fed HFD+WB reduced the fatty infiltration of hepatocytes in the MS+Lj group (Figure 4a).

Transaminase levels were measured in plasma; increases in AST and ALT activities of 68% and 60% were observed in the MS group compared to the Control group. By contrast, in the MS+Lj group, the AST and ALT activities did not show differences from the Control group (Figure 4b).

### 3.5. Effect of Lj CRL1231 on Intestinal FE Activity and Oxidative Status

The intestinal feruloyl esterase (IFE) activity decreased by 41% in the MS group (733.01 ± 82.91 U/g) compared to the Control (1255.61 ± 50.28 U/g). When *Lj* CRL1231 was administered, the IFE activity increased by 38% compared to the MS group (1011.00 ± 187.11 U/g).

Liver GPx activity showed a reduction of 10% in the MS group (30.51 ± 0.74 U/g) compared to the Control group (34.16 ± 0.98 U/g). Similarly, liver GR activity showed a 15% reduction in the MS group (45.91 ± 1.95 U/g) compared to the Control group (53.92 ± 1.31 U/g). In the MS+Lj group, GPx activity was 33.93 ± 1.80 U/g, and GR activity was 70.95 ± 2.99 U/g, showing increases of 11% and 54%, respectively, compared to the MS group.

Plasma lipoperoxides (TBARS) were 47% higher in the MS group (47.95 ± 4.55 nmol/g) compared to the Control group (32.43 ± 6.61 nmol/g), and these levels were significantly reduced (*p* < 0.05) in the MS+Lj group (32.31 ± 5.91 nmol/g), even reaching the values of the Control group.

### 3.6. Effect of Lj CRL1231 on Plasma Lipid and Parameters in Assessing Cardiovascular Risk

**Figure 5:**
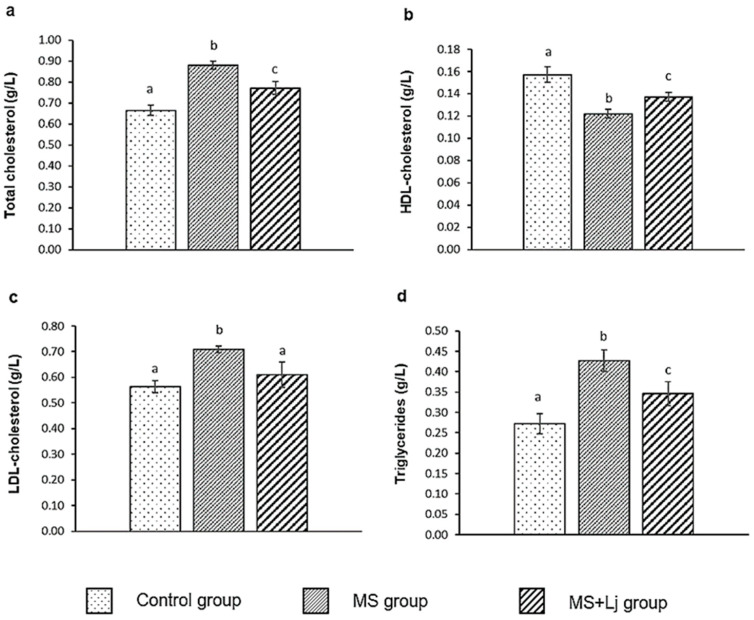
Plasma lipids (**a**) total cholesterol (g/L), (**b**) HDL-cholesterol (g/L), (**c**) LDL-cholesterol (g/L), and (**d**) triglycerides (g/L). Data are expressed as mean ± SE (standard error), *n* = 8 per group. Values with different letters are significantly different (*p* < 0.05). Control: mice receiving normal diet; MS (Metabolic syndrome): mice receiving HFD+WB diet; MS+Lj: mice receiving HFD+WB diet and *L. johnsonii* CRL1231.

Plasma lipids were measured in all groups of mice tested (Figure 5). Total cholesterol increased 32% in the MS group in 14 weeks of HFD+WB feeding compared to the Control group. When *Lj* CRL1231 was administered, a 12% decrease in total cholesterol was observed in the MS+Lj group compared to the MS group (Figure 5a).

The HDLc values were 22% lower in the MS mice compared to the Control mice. The MS+Lj group showed 12% higher HDLc levels than the MS group (Figure 5b).

The LDLc increased by 27% in the MS group compared to the Control group. However, when the mice received *Lj* CRL1231 together with HFD+WB, the LDLc levels were similar to the Control group (Figure 5c).

Plasma triglyceride concentration was 57% higher in the MS mice compared to the Control group. With probiotic supplementation, hypertriglyceridemia was reduced by 19% in the MS+Lj group compared to the MS group (Figure 5d).

An increase in cardiovascular risk indicators was found in the MS group compared to the Control group (approximately a 2-fold increase). Values of risk indicators were reduced in the MS+Lj group with respect to the MS group, and even CRI-II was similar to the Control group (Table 2).

### 3.7. Effect of Lj CRL1231 on Glucose Metabolic Disorders

Animals receiving HFD+WB for 14 weeks (MS group) had fasting glucose increased by 96.5% compared to the Control. The administration of *L. johnsonii* caused a 31.5% decrease in blood glucose values in the MS+Lj group compared to the MS group (Table 2).

Insulin levels were 46.5% higher in the MS group; however, the administration of *Lj* CRL1231 managed to keep insulin concentrations in the MS+Lj group close to the Control group value (Table 2).

The HOMA-IR index was 3 times higher in the MS group compared to the Control group, while the MS+Lj group did not show significant differences from the Control (Table 2).

**Figure 6:**
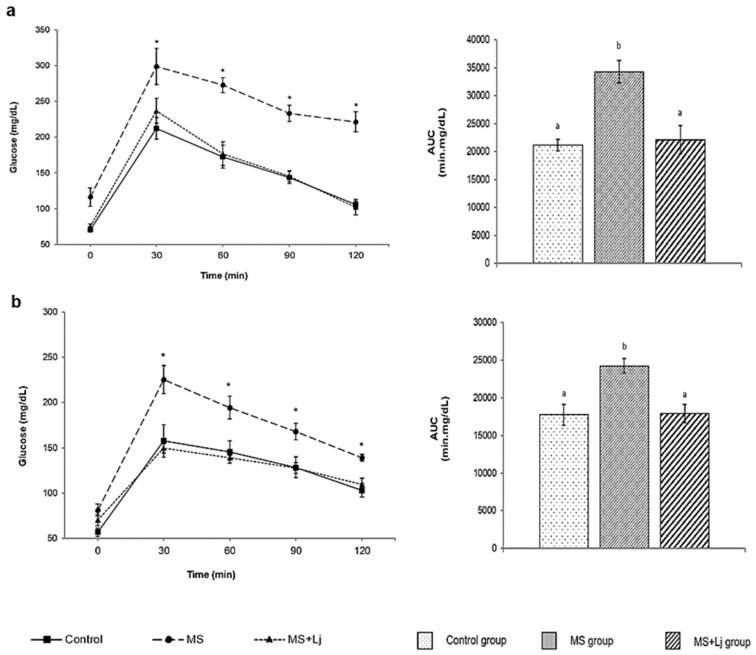
Oral glucose and sucrose tolerance test. (**a**) Glucose tolerance curves at 14 weeks of feeding (left side) and the corresponding areas under the curve (AUC) (right side). (**b**) Sucrose tolerance curves at 14 weeks of feeding (left side) and the corresponding areas under the curve (AUC) (right side). Data represent mean ± SE (standard error) of *n* = 8 mice. Statistical differences were determined using Tukey’s test with *p* < 0.05. In the curves on the left side, the asterisks (*) indicate statistical differences concerning the Control group. In the figures on the right side, values with different letters differ significantly.

The oral glucose tolerance test (OGTT) at 14 weeks of feeding showed that the MS group presented significantly higher glucose levels at all points of the tolerance curve compared to the rest of the groups evaluated, with values higher than 200 mg/dL after 2 h of oral glucose overload, which is an indication of insulin resistance. The area under the curve was 62% higher in the MS group. The MS+Lj group did not differ from the Control group in postprandial glucose values (Figure 6a).

The antihyperglycemic effect of *L. johnsonii* CRL123 was evaluated by performing the oral sucrose tolerance test (OSTT) at week 14. The results showed a significant increase in blood glucose concentration at each point of the curve in the MS group (with an increment of 37% in the area under the curve), while there were no significant differences between the MS+Lj and the Control groups (Figure 6b). Concerning the α-glucosidase inhibitory capacity, *Lj* CRL1231 inhibited α-glucosidase activity by 75.67%, suggesting that the antihyperglycemic effect observed in vivo in the OSTT may be associated with the administration of this probiotic strain.

### 3.8. Influence of Lj CRL1231 on Metabolites from FA and SCFA Production in Colon Contents

The FA-derived metabolites in the colon contents of the mice were determined semi-quantitatively by mass spectrometry and chromatograms (Supplementary Materials, Figure S2). Dihydroferulic acid (DHF), 3,4-dihydroxyphenylpropionic acid (DHPPA), and 3-hydroxyphenylpropionic acid (HPPA) were detected in the three groups of mice tested (Table 3 and Supplementary Materials, Figure S1). A lower abundance of these metabolites was observed in the MS group compared to the Control. However, in the MS+Lj group, an increase was observed compared to the MS group, mainly in HPPA, which turned out to be the most abundant metabolite. On the other hand, FA and benzoic acid were not detected (Table 3).

The concentrations of SCFAs in the large intestine contents of the mice are shown in Table 4. A significant reduction (40%) in total SCFA levels was observed in the MS group compared to the Control. By contrast, the MS+Lj group exhibited a 33% increase in total SCFA concentration compared to the MS group. Specifically, the MS+Lj group showed a 1.2-fold increase in acetic acid, a 2-fold increase in propionic acid, and a 3-fold increase in butyric acid compared to the MS group. Notably, in the MS+Lj group, butyric acid concentrations were comparable to those in the Control group, while propionic acid levels exceeded those observed in Control mice (Table 4).

### 3.9. Modulation of IM by Lj CRL1231 and Diet

Alpha diversity of the intestinal microbial communities was estimated in mice from the Control, the MS, and the MS+Lj groups, and no significant differences were found in the Chao 1 index between the three groups evaluated. There was a tendency towards a lower richness of the bacterial community in the animals that were fed with the HFD+WB (MS and MS+Lj groups). The number of observed species (OTUs) was significantly lower in the MS group compared to the Control group, but this value was partially reversed with the administration of *Lj* CRL1231 (MS+Lj group) (Table 5). Regarding the Shannon index (diversity estimator) and phylogenetic diversity (PD whole tree), no statistically significant differences were observed between all groups evaluated (Table 5).

**Figure 7:**
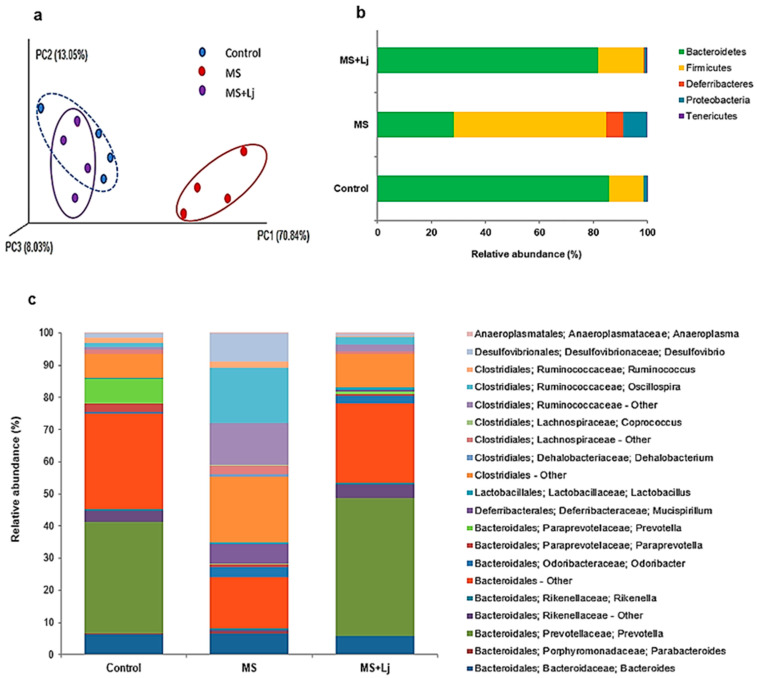
Metagenomic analysis from DNA obtained from fecal samples of mice fed for 14 weeks with normal diet (Control group), HFD+WB (MS group), and HFD+WB supplemented with *L. johnsonii* CRL1231 (MS+Lj group). (**a**) Weighted UniFrac-based PCoA of gut microbiota (beta diversity) via 16S rRNA sequencing. (**b**) Relative abundance of intestinal bacterial communities grouped by phylum. (**c**) Relative abundance of intestinal bacterial communities grouped by family and genus.

Beta diversity was calculated using UniFrac analysis and estimated distances between the samples of the three groups (Figure 7a). The three-dimensional scatter plot generated using principal coordinate analysis (PCoA) clustered the Control and the MS+Lj IM communities, separating them from the IM of the MS mice (Figure 7a).

The metagenomic analysis showed the relative abundance of the intestinal bacterial community in the groups of mice under study. Between the three groups of mice evaluated, the Bacteroidetes and Firmicutes phyla were predominant (Figure 7b). However, the proportions of these bacterial phyla were modified according to the diet administered. Bacteroidetes were less abundant in the MS group (28.20%) compared to the Control group (85.80%), and the proportion was similar to the Control when *Lj* CRL1231 was administered in the MS+Lj group (81.85%). A high abundance of Firmicutes was observed in the MS group (56.55%) compared to the Control group (12.75%), while in the MS+Lj group, the relative abundance of Firmicutes was closer to that of the Control (16.65%) (Figure 7b).

The Bacteroidetes phylum includes microorganisms belonging to the Bacteroidales order. Within this group, a population whose family and sex were not identified (Bacteroidales—Other: Control, 29.70%; MS, 16%; MS+Lj, 24.70%) was the dominant. The *Prevotella* genus (Prevotellaceae family) showed significant differences between the groups of mice evaluated since it was absent in the MS group (0%), but its proportion was high in the Control (35%) and the MS+Lj (43%) groups. The *Bacteroides* genus had a relative abundance of about 6% in all groups. On the other hand, the Rikenellaceae family experienced a reduction, showing a low abundance in the MS group (0.7%) compared to the Control group (3.35%). However, the administration of *Lj* CRL1231 increased the proportion of this bacterial group in the MS+Lj mice group (4.15%) (Figure 7c).

The Firmicutes phylum included members of the order Clostridiales and Lactobacillales. Among the Clostridiales, the Ruminococcaceae family prevailed, and the *Oscillospira* genus was the most relevant; it showed great abundance in the MS group (17.35%) compared to the Control (1.40%) and the MS+Lj groups (2.40%). In addition, a group of unclassified Clostridiales stood out (Clostridiales—Other: Control, 7%; MS, 20%; MS+Lj, 10.25%). Further, even in smaller proportions, the Lachnospiraceae family was modified according to the diet administered to each experimental group (Control, 1.30%; MS, 3%; MS+Lj, 0.60%). Regarding the Lactobacillales family, a higher abundance of the *Lactobacillus* genus was found in the MS+Lj group (0.80%) compared to the Control (0.20%) and the MS (0.30%) groups (Figure 7c).

Regarding the Proteobacteria phylum, the *Desulfovibrio* genus (Desulfovibrionaceae family) was more abundant in the MS group (8.85%) compared to the Control (1.25%), and there was a reduction in the MS+Lj group, where less abundance was observed (0.6%) (Figure 7c).

Additionally, changes in bacterial counts of the fecal microbiota of each experimental group were analyzed by qPCR (Table 6). No significant differences were found in the gene copy numbers of the Bacteroides. In the MS group, there was an increase in the gene copy numbers of *Lactobacillus* and Enterobacteriaceae compared to the Control group (8% and 30%, respectively). With the administration of *Lj* CRL1231 (MS+Lj group), no changes were observed concerning the MS group, although there was a tendency towards a greater amount of *Lactobacillus* in the MS+Lj group. On the other hand, the gene copy numbers of *Bifidobacterium* were reduced in the MS group compared to the Control group (a 20% reduction). Nevertheless, probiotic supplementation in the MS+Lj group allowed these values to be normalized (with the number of *Bifidobacterium* similar to the Control group) (Table 6).

## 4. Discussion

Metabolic syndrome (MS) is a complex pathophysiological state with a prevalence of 25% of adults worldwide [26]. Modern dietary habits that increasingly include high-fat foods can lead to the development of MS, characterized by obesity/increased abdominal fat, hypertension, insulin resistance, and dyslipidemia, and are associated with the development of T2D, CVD, and NAFLD. MS can lead to a 2-fold increase in the risk of CVD and cerebrovascular disease and a 1.5-fold increase in the risk of all-cause mortality, constituting a major public health challenge [27].

In the present work, mice undergoing a daily intake of HFD+WB for 14 weeks showed not only an increase in body weight gain but exhibited a greater ability to transform grams of food consumed into grams of body mass (increased FER); this was directly reflected in the high adiposity index of the animals in the MS group, in whose histological sections of epididymal adipose tissue the majority presence of large adipocytes was observed. Furthermore, a positive relation was found between the adiposity index and leptin levels, which increased in the mice with MS.

Adipose tissue is an active and complex endocrine organ that secretes molecules and plays key roles in inflammation, immune response, appetite regulation, vascular events, reproductive functions, and insulin sensitivity. An excess of adipose tissue, particularly in the visceral compartment, is associated with morbidity and complications of MS due to the increased production of inflammatory molecules (cytokines and adipokines) [28]. Studies have shown that obese individuals secrete higher levels of some cytokines (leptin, TNF-α, IL6), promoting inflammation, and probiotics may help regulate these bioactive compounds released by adipose tissue [29].

In recent years, modulation of the IM through the consumption of probiotic bacteria has emerged as a promising strategy for preventing and mitigating MS. Increasing evidence has suggested that gut microorganisms produce bioactive compounds capable of regulating appetite, contributing to energy homeostasis, and reducing adipose tissue accumulation [7]. Oral administration of *Lj* CRL1231 exerted beneficial effects in the MS+Lj group for 14 weeks, as evidenced by reduced BWG, FER, and adiposity index. Moreover, a histological analysis of the epididymal adipose tissue showed that *Lj* CRL1231 preserved a tissue architecture comparable to that of the Control animals, with smaller and more uniformly distributed adipocytes. Lee et al. (2022) [30] investigated strains with anti-adipogenic potential and reported that *L. johnsonii* 3121 could function as a probiotic bacterium, preventing fat accumulation by regulating adipogenesis-related markers. The inhibition of adipocyte differentiation was proposed as a potential mechanism to prevent obesity.

Additionally, the *Lj* CRL1231 strain ameliorated hyperleptinemia, significantly lowering leptin levels in the mice fed with the HFD+WB. Cano et al. (2013) [25] suggested that, in diet-induced obesity models, probiotic supplementation may help restore leptin sensitivity or functionality, promoting a more balanced distribution of adipose tissue and preventing triglyceride accumulation in peripheral organs.

In addition to being positively correlated with different MS parameters, leptin participates in the promotion, recruitment, and proliferation of pro-inflammatory immune cells [29].

The chronic inflammatory state that characterizes obesity was reflected in the MS mice by increased TNF-α, IFN-γ, and IL-6 plasma levels. However, the administration of *Lj* CRL1231 improved the inflammatory profile in the MS+Lj group by reducing pro-inflammatory cytokines and increasing IL-10 (anti-inflammatory). A recent study demonstrated that *L. johnsonii* UMNLJ22 can attenuate induced colitis in mice. A reduction in the infiltration of the immune cells and the secretion of pro-inflammatory cytokines was associated with these protective effects [31]. Another study reported that *L. johnsonii* 6084 significantly decreased the expression of serum inflammatory cytokines, IL-1β and TNF-α, in septic mice [32].

Some pathological conditions, such as obesity, generate a disturbance of the IM (known as dysbiosis) and a subsequent deterioration of the intestinal barrier function, allowing some microbial molecules (such as lipopolysaccharide (LPS) and peptidoglycans) to cross the barrier and pass into the bloodstream, triggering systemic pro-inflammatory signaling that, in turn, causes MS related metabolic disturbances, such as NAFLD, insulin resistance, and hyperglycemia [28,33]. NAFLD is the hepatic manifestation of MS. It was shown that *Lj* CRL1231 can prevent fatty infiltration in the liver of mice fed with HFD+WB by improving the visible intracellular vacuolization that marked lipid accumulation and hepatic steatosis. AST and ALT are indicators of liver function, and their serum levels increase when the hepatocyte structure is damaged [34,35]. Oral administration of *Lj* CRL1231 not only reduced the abundant deposit of lipid vesicles in the liver but decreased the high levels of AST and ALT transaminases in the MS+Lj group, suggesting that this strain would help to protect against the liver damage characteristic of MS. Consistent with our findings, Xin et al. (2014) [35] demonstrated that *L. johnsonii* BS15 may prevent diet-induced NAFLD through adjusting the IM, improving mitochondrial dysfunction, and reducing the intestinal permeability, level of serum LPS, insulin resistance, and inflammation.

During the obesity process accompanying MS, excess free radicals are produced, leading to lipid peroxidation, oxidative stress, and the resulting inflammation [36]. We observed that oral administration of *Lj* CRL1231 reduced the levels of plasmatic lipoperoxides (TBARS) and increased the hepatic GPx and GR activities relative to the MS group. Considering that the administered bacterial strain has FE activity and that the diet (HFD+WB) has WB fibers in its composition, which is rich in ferulates, dietary supplementation with *Lj* CRL1231 that acts on bran fiber favors the intestinal FE activity, increasing the release and metabolism of FA. FA has potent antioxidant activity and has been widely used to prevent diseases associated with reactive oxygen species (ROS), such as MS, T2D, CVD, and cancer [37]. Previous studies have shown that the administration of encapsulated *Lj* CRL1231 improves the oxidative status of HFD-induced obese mice with a marked increase in hepatic GPx and GR activities [13]. Another study found that treatment with *L. johnsonii* BS15 can ameliorate oxidative stress in liver tissue [35]. Additionally, Mahmoud et al. (2020) [38] reported that FA decreased the concentrations of ROS, malonyldialdehyde (MDA), and nitric oxide (NO) in the liver of rats, ameliorating liver damage.

Concentrations of plasma lipids observed in the mice fed with HFD+WB reflect the characteristic dyslipidemia of MS. Results obtained for the MS+LJ group showed that the supply of *Lj* CRL1231 could prevent hypercholesterolemia and hypertriglyceridemia induced by HFD+WB intake. In addition, the *Lj* CRL1231 oral administration allowed for the maintenance of higher levels of HDLc in the mice fed with the HFD+WB. The strategy of combining a probiotic strain with FE activity and esterified phenolic compounds (present in the diet containing WB) may be effective in preventing MS dyslipidemia, as also indicated by the results obtained by Teixeira et al. (2021) [39], which suggested that sterol regulatory element binding protein 1 (SREBP-1) plays a fundamental role in the regulation of fatty acid metabolism in the liver of rats fed with *L. johnsonii* N6.2 (with cinnamyl esterase activity) and blueberry (with phenolics). In another study, Qing et al. (2017) [40] found an increase in HDLc in broiler chickens with subclinical necrotic enteritis (SNE) after treatment with *L. johnsonii* BS15, suggesting that BS15 supplementation may help to remit dyslipidemia caused by SNE. FA released from the diet exerts beneficial effects in improving lipid metabolism; however, the underlying mechanisms remain unclear. In a recent study, Gao et al. (2022) [14] highlighted FA as a potential alternative nutritive agent targeting long-chain acyl-CoA synthase 1 (ACSL1) to improve lipid metabolism in diabetic mice, as metabolomics results untargeted suggested the participation of FA in the regulation of lipid metabolism, identifying through targeted fishing ACSL1 as the target of FA. This event initiates a cascade of reactions that ultimately inhibit the synthesis of triglycerides and cholesterol.

MS is the underlying cause of devastating diseases like CVD. Cardiovascular risk indicators have an elevated predictive capacity for those individuals who present the pathologies associated with MS, such as obesity, insulin resistance, and T2D, all conditions linked to the development of atherogenic CVD, in which dyslipidemia contributes significantly to its pathogenesis [41]. *Lj* CRL1231 contributed to reducing cardiovascular risk indices by improving lipid homeostasis in the mice fed HFD+WB, a beneficial feature also reported by our group for another probiotic strain using the same animal model and diet [13].

*Lj* CRL1231 improved the parameters related to glucose metabolism (glycemia, insulin, HOMA-IR). It was also observed that it attenuated glucose intolerance (OGTT) in the mice fed with HFD+WB, suggesting that administration of this strain with FE activity improves insulin sensitivity and prevents hyperglycemia during the development of MS. Similar results were found in another study with *L. johnsonii* BS15 supplementation that significantly reversed the increase in HOMA-IR, serum insulin, and glucose levels in NAFLD mice with a dose-dependent effect [35]. The increased release of FA by *Lj* CRL1231 could be one of the reasons for the observed glucometabolic effects. Some studies have shown that FA supplementation significantly reduces the increase in fasting glucose induced by an HFD [37]. According to a previous study, the acid anti-diabetic activity is associated with the improvement of insulin sensitivity and hepatic glycogenesis—by phosphorylating and inhibiting glycogen synthase kinase-3β (Gsk3β)—as well as suppressing gluconeogenesis—by phosphorylating transcription factor-O1 (Foxo1)—aside from inhibiting the negative regulators of insulin signaling [42].

In recent years, some lactic acid bacteria strains have been reported to possess α-glucosidase inhibitory activity and could alleviate the effects of T2D without adverse side effects [43]. Performing the OSTT was an indirect way of evaluating the inhibitory effect on α-glucoside activity by *Lj* CRL1231, since the results showed that oral administration of this strain reduced postprandial glucose levels in the MS+Lj mice that received a sucrose overload. Additionally, an in vitro assay demonstrated that *Lj* CRL1231 can inhibit α-glucosidase. The α-glucosidase enzyme is a hydrolase attached to the brush border of intestinal cells that hydrolyzes complex carbohydrates into glucose for absorption by the intestine. Inhibiting the intestinal α-glucosidase activity leads to reduced disaccharide (sucrose) hydrolysis, which, in turn, can reduce glucose release and absorption, thus lowering blood glucose levels [44].

Many studies have explored natural dietary approaches to mitigate the effects of MS. However, harnessing beneficial metabolites, like FA and its derivatives, within the gut remains difficult due to the complex structure of plant cell walls and poor bioavailability. The great difficulty that FA presents is that, although it is naturally present in foods, it is in a large proportion covalently bound by ester bonds to the arabinoxylan polysaccharides of the cell wall, lignin, and other polymers (fiber components). Its release, absorption, and metabolization in the gastrointestinal tract are limited, and therefore, its ability to induce beneficial changes is reduced. For this reason, we propose using *Lj* CRL1231 with FE activity as a preventive agent and/or new adjuvant therapeutic for mice who develop MS.

The administration of *Lj* CRL1231 benefits the intestinal status in mice that receive HFD+WB since it causes changes in the microbiota, modifies IFE activity, and directly influences the production of FA-derived metabolites and the levels of SCFAs. The IFE activity allows for breaking the ester bond that joins FA with polysaccharides of the wall in the WB fiber. In the Control group animals, ester bond cleavage to release FA is possible in the colon through the action of fecal microbial xylanases and esterases [18]. However, the release of FA was altered in the mice fed HFD+WB, and the IFE activity was reduced in the MS group. Nevertheless, when *Lj* CRL1231 was administered, the intestinal enzymatic release of FA increased. A recent study has shown that enzymatic processing (with FE) of high-fiber bread can increase FA bioavailability and improve human vascular function [18]. Previous studies have demonstrated that microencapsulated FE-producing *Lj* CRL1231 can increase intestinal FE activity in obese mice to values similar to the Control group [13]. As mentioned, the host cannot efficiently use the FA combined with arabinoxylan in cereals (insoluble FA). However, this insoluble FA in cereals is hydrolyzed by the IM and released to blood at a relatively slow rate, so it has a more stable and efficient bioavailability than the intake of pure FA [45]. The *Lj* CRL1231 administration promoted intestinal FE activity and in situ release of FA, increasing its bioavailability compared with the FA present in a pure commercial formula.

The role of the IM in the biotransformation of dietary phytochemicals, including FA, is well established. Metabolites of FA produced by the IM can be rapidly absorbed and enter the bloodstream [46,47,48]. In our study, we found that the production of colonic metabolites of FA (HPPA, DHPPA, and DHF) was greater in the animals that received *Lj* CRL1231, which reaffirms the increase in IFE activity in the animals that received the probiotic. Other authors have identified the colonic metabolites of phenolic acids, studying anaerobic degradation in vitro (using human feces). They have demonstrated that biotransformation of the 8-O-4-diferulic acid dimer (C-O-C bond) began with the release of FA, which was converted to hydroxylated phenylpropionic acids, especially to the formation of HPPA. At the same time, the 5-5-diferulic acid dimer (C-C bond) underwent a series of demethylations and reductions that resulted in the formation of DHPPA (also called dihydrocaffeic acid), caffeic acid, FA, or DHF [46,47,48].

Acetate, propionate, and butyrate are the most abundant SCFAs produced by the IM from the fermentation of dietary fiber. In a recent study, Wang et al. (2021) [49] proposed that modulation of the IM and SCFAs is a pathway to suppress the development of MS, suggesting that the protective effects of several *Lactobacillus* strains are mediated in part by the recovery of SCFA production. In this sense, we observed that, although the MS mice showed an expected reduction in total SCFAs in the colon, probiotic supplementation with *Lj* CRL1231 managed to increase the concentration of SCFAs in the mice fed with HFD+WB. Higher levels of intestinal SCFAs have beneficial effects against obesity and T2D [50]. SCFAs can also improve intestinal barrier integrity, regulate discomfort, and promote human health [51]. Preclinical and clinical studies have revealed that SCFAs can increase the secretion of intestinal hormones, such as GLP-1, GLP-2, and PYY (reducing appetite), and decrease the level of systemic inflammatory cytokines by disrupting lipid breakdown, and therefore increase energy consumption and lipid oxidation to alleviate obesity [52].

While the structure of the IM is altered by various factors during the development of metabolic diseases, such as diet and lifestyle, counteracting the IM imbalance (dysbiosis) with probiotics is key to reducing metabolic disorders [49]. In this sense, we found that *Lj* CRL1231 induced favorable changes in the IM structure of the mice fed HFD+WB, such that the effects of the diet in these animals were much less harmful than in their peers that followed the same type of feeding but without probiotic supplementation.

An analysis of bacterial richness (Chao 1 index) revealed a tendency towards a reduction in the IM richness in the mice fed the HFD+WB (MS and MS+Lj groups). When the number of OTUs was observed, we noticed that the administration of *Lj* CRL1231 marked a positive change in the mice fed HFD+WB, preventing the loss in the number of species observed in the IM of the MS+Lj mice, modulating a healthy change in the microbiota. Additionally, when we measured beta diversity, the bacterial communities of the MS and the MS+Lj mice were grouped separately, and the MS+Lj group was similar to the Control group. Teixeira et al. (2021) [39] analyzed the IM of rats fed with HFD supplemented with *L. johnsonii* N6.2 and blueberries, showing that beta diversity was significantly modified by *L. johnsonii* N.6 and observed a reduction of alpha diversity. However, rats with a low-calorie diet supplemented with *L. johnsonii* N6.2 and blueberries found an increase in alpha diversity. Given this, the supplementation with *L. johnsonii* would serve as an adjuvant to alleviate the intestinal and systemic alterations caused by excessive fat intake; however, it is necessary to apply an adequate diet in individuals who suffer from MS. There are reports in humans indicating that the intestinal bacterial diversity was low in obese men affected by MS but increased after a microbiota transfer capable of improving insulin sensitivity [53]. Nevertheless, other studies do not show the differences in bacterial diversity between normal subjects and those with MS [54].

Interpreting the variations observed between the experimental groups concerning the type and function of bacteria that constitute the IM is crucial for understanding the observed effects. Obesity changes the IM, affecting the two main phyla in human and mouse intestines; it is characterized by an increase in Firmicutes (F) and a reduction in Bacteroidetes (B) [55]. Firmicutes are generally referred to as ‘bad microbes’, disrupting glucose and lipid metabolism, so a high F/B ratio correlates with an increased risk of T2D [56]. In this research, we found that the F/B ratio increased in the MS group, which is consistent with previous research in obesity models [55,57]. The administration of *Lj* CRL1231 was able to counteract the changes at the phylum level, normalizing the F/B ratio through the relative abundances similar to the Control group. Interestingly, Tian et al. (2022) [51] found that the FA treatment modulates the microbial taxonomic profile in HFD-fed mice, restoring the F/B ratio. Our findings are consistent with those reported by Wang et al. (2017) [58], who mentioned that the low percentage of abdominal fat in broiler chickens treated with *L. johnsonii* BS15 can be attributed to the changes induced in the IM, where they observed an improvement in the population of Bacteroidetes and *Lactobacillus*, with a reduction in Enterobacteriaceae and F/B ratio.

We observed that the administration of *Lj* CRL1231 increased the gene copy numbers of *Bifidobacterium*, which were reduced in the MS group, but could not normalize the numbers of Enterobacteriaceae increased by the consumption of HFD+WB. We also detected that supplementation with *Lj* CRL1231 tended to increase the numbers of *Lactobacillus*. No effect was observed from the HFD+WB or the administration of *Lj* CRL1231 on the copy numbers of *Bacteroides*. In line with our results, earlier studies have found that *Bifidobacterium* was negatively correlated with obesity [59,60]. *Bifidobacterium*, a beneficial microbial species capable of producing lactic and acetic acids, can reduce the intestinal pH, inhibit the growth of detrimental bacteria, and maintain intestinal health [36,59].

Interestingly, the MS group of mice exhibited an increased relative abundance of Proteobacteria, particularly the genus *Desulfovibrio*, along with higher copy numbers of *Lactobacillus.* Other authors have similarly verified that the IM in T2D was composed of a greater proportion of sulfate-reducing bacteria, especially *Desulfovibrio* and *Lactobacillus* [56,61]. The *Lj* CRL1231 supplementation had a positive effect and prevented the increase of *Desulfovibrio* in the mice fed HFD+WB. In vitro and in vivo studies with diabetic rats have shown that feruloylated oligosaccharides and FA from WB suppressed *Desulfovibrio* growth, which is consistent with our current study [56,62]. In line with this, a recent study found that the levels of *Bacteroidetes* and *Proteobacteria* changed in septic mice, while the probiotic *L. johnsonii* 6084 restored the balance of the IM and reduced the loss of microbial diversity and richness [33].

A relative abundance analysis showed a higher proportion of the *Ruminococcaceae* family (genus *Oscillospira*) and *Lachnospiraceae* in the MS mice, while the MS+Lj mice had a lower abundance. A reduced abundance of *Ruminococcaceae* has been reported to favor intestinal homeostasis, although many members of this family are SCFA producers [7]. On the other hand, the *Lachnospiraceae* family has been associated with diet-related obesity in humans [63]. Several members of *Lachnospiraceae* can suppress the growth of SCFA-producing bacteria and are associated with metabolic disorders and cancer. However, the treatment with a probiotic mixture of *L. plantarum* has been able to reverse these changes in HFD-fed mice [59].

The administration of *Lj* CRL1231 yielded optimistic results, as it induced positive changes in the IM of mice fed HFD+WB, balancing the microbial population altered by the diet and allowing for the greater production of beneficial FA metabolites at the intestinal level. The results obtained in the present research suggest that the IM intervention with *Lj* CRL1231 allowed for the attenuation of metabolic, oxidative, and systemic alterations present in MS. While additional research is required to determine the most relevant bacterial species and the biochemical mechanisms linking intestinal changes to health improvements, our results provide a foundation for developing an effective dietary intervention against MS.

## 5. Conclusions

The co-administration of *L. johnsonii* CRL1231 and wheat bran represents a novel and promising strategy to mitigate the multifactorial consequences of metabolic syndrome. In the MS+Lj group, intestinal feruloyl esterase activity increased by 38% compared to the MS group, leading to the enhanced release of ferulic acid and its metabolites (dihydroferulic, dihydroxyphenylpropionic, and hydroxyphenylpropionic acids). Concurrently, *Lj* CRL1231 modulated the gut microbiota, showing a 3.4-fold decrease in Firmicutes and a 2.9-fold increase in Bacteroidetes, with a 93% reduction in *Desulfovibrio*. By contrast, *Bifidobacterium* levels rose significantly (6.58 log CFU/g), nearly reaching those of the Control group.

These microbial shifts promoted the enhanced fermentation of dietary fiber, resulting in a 33% increase in total SCFAs, specifically acetic (1.2-fold), propionic (2-fold), and butyric acids (3-fold), compared to the MS group. Collectively, these changes translated into notable physiological benefits in the MS+Lj group, including a 26% reduction in the adiposity index, a 12% increase in HDL cholesterol, a 19% decrease in triglycerides, normalization of glucose and insulin levels, with a 2-fold reduction in HOMA-IR, improved inflammatory profile (decreased TNF-α, IFN-γ, and IL-6 by 3-, 5-, and 2-fold, respectively, and a 2-fold increase in IL-10), restoration of liver function markers, and prevention of oxidative stress, with TBARS levels comparable to the Control.

The beneficial effects observed cannot be attributed to a single mechanism but rather to a synergistic interplay between the probiotic and the fiber source. *Lj* CRL1231 exhibits feruloyl esterase activity and can metabolize wheat bran, suggesting a prebiotic–probiotic interaction that supports the development of future synbiotic formulations. Furthermore, *Lj* CRL1231 showed a significant intestinal α-glucosidase inhibitory activity (75.67%), improving postprandial glucose control in sucrose tolerance tests, an important finding given the close link between MS and type 2 diabetes.

Altogether, these results highlight the role of gut microbiota modulation in promoting metabolic health. The specific contribution of *Lj* CRL1231 lies in enhancing the release and biotransformation of ferulic acid, increasing fiber fermentation, and boosting SCFA production. These combined effects support a promising dietary approach for mitigating the risks associated with metabolic syndrome. However, further studies are needed to elucidate the precise metabolic pathways involved and to validate these findings in clinical settings.

## Figures and Tables

**Table 1:** Experimental groups. Control: mice receiving normal diet; MS (Metabolic syndrome): mice receiving HFD+WB diet; MS+Lj: mice receiving HFD+WB diet and *L. johnsonii* CRL1231.

	Experimental Groups		
	Control	MS	MS+Lj
Gavage administration	100 µL of water	100 µL of water	100 µL of *L. johnsonii* CRL1231 suspension (final dose: 10^8^ cells/day)
Diet	* ND: Normal diet. Calorie content: 3.1 Kcal/g (6.5% oil vegetable-derived Kcal).	* HFD+WB: High-fat diet supplemented with wheat bran to 7% (*w*/*w*). Calorie content: 5.1 Kcal/g (with 60% lard-derived Kcal).	* HFD+WB: High-fat diet supplemented with wheat bran to 7% (*w*/*w*). Calorie content: 5.1 Kcal/g (with 60% lard-derived Kcal).
Drinking water	*ad libitum*	*ad libitum*	*ad libitum*

* ND and * HFD+WB diets supplied approximately 0.60 mg of hydroxycinnamates from WB/day/mouse.

**Table 2:** Cardiovascular risk indicators, plasma glucose, insulin, and HOMA-IR.

	Control	MS	MS+Lj
Cardiovascular risk indices			
*AC	3.19 ± 0.43 ^a^	6.20 ± 0.53 ^b^	4.62 ± 0.54 ^c^
**CRI-I	4.23 ± 0.62 ^a^	7.21 ± 0.25 ^b^	5.62 ±0.41 ^c^
**CRI-II	3.59 ±0.33 ^a^	5.82 ± 0.24 ^b^	4.45 ± 0.40 ^a^
***AIP	0.23 ± 0.05 ^a^	0.53 ± 0.04 ^b^	0.40 ± 0.05 ^c^
Glucose (mmol/L)	3.97 ± 0.47 ^a^	7.80 ± 0.92 ^b^	5.34 ± 0.55 ^c^
Insulin (µU/mL)	8.70 ± 0.70 ^a^	12.60 ± 0.86 ^b^	8.96 ± 0.97 ^a^
HOMA-IR	1.54 ± 0.28 ^a^	4.37 ± 0.42 ^b^	2.13 ± 0.77 ^a^

Data are expressed as mean ± SE (standard error), *n* = 8 per group. Values with different superscript letters in the same row are significantly different (*p* < 0.05). Control: mice receiving normal diet; MS (Metabolic syndrome): mice receiving HFD+WB diet; MS+Lj: mice receiving HFD+WB diet and *L. johnsonii* CRL1231. *AC: Atherogenic coefficient = [(Total cholesterol—HDLc)/HDLc]; **CRI: Castelli risk index: **CRI-I = [Total cholesterol/HDLc]; **CRI-II = [LDLc/HDLc]; ***AIP: Atherogenic index of plasma = [Log (Triglyceride/HDL)].

**Table 3:** Semi-quantitative determination of FA-derived metabolites in colon contents of mice by HPLC-MS.

			Control	MS	MS+Lj
Compound	Rt (min)	Molecular Ion [M − H]^−^ (*m/z*)			
FA	12.55	193.0	−	−	−
DHF	8.26	195.1	+	+	++
DHPPA	7.36	181.1	++	+	++
HPPA	8.56	165.1	+++	+	++++
BA	13.2	121.0	−	−	−

The mass/charge ratios (*m*/*z*) and retention times (RT) for each compound are shown. FA: ferulic acid; DHF: dihydroferulic acid; DHPPA: dihydroxyphenylpropionic acid; HPPA: hydroxyphenylpropionic acid; BA: benzoic acid. The metabolites detected are shown for the Control, the MS, and the MS+Lj groups *(n* = 4 per group). (−): metabolite not detected (+): metabolite detected. More signs (++, +++, or ++++) indicate a greater area of the peaks in the spectrum.

**Table 4:** Concentration of short-chain fatty acids (SCFAs) produced in colon contents at week 14 of feeding.

	Control	MS	MS+Lj
SCFA (nmol/g fecal content)			
Acetic acid	12.24 ± 0.15 ^a^	7.59 ± 0.45 ^b^	9.05 ± 0.58 ^c^
Propionic acid	4.45 ± 0.24 ^a^	2.71 ± 0.09 ^c^	5.64 ± 0.40 ^b^
Butyric acid	1.40 ± 0.15 ^a^	0.52 ± 0.04 ^b^	1.50 ± 0.18 ^a^
TOTAL SCFAs	18.09 ± 0.18 ^a^	10.82 ± 0.19 ^c^	16.19 ± 0.39 ^b^

Data represent mean ± SE (standard error) of *n* = 8 mice per group. Values with different superscript letters in the same row differ significantly (*p* < 0.05). Mice received normal diet (Control group), HFD+WB (MS group), HFD+WB supplemented with *L. johnsonii* CRL1231 (MS+Lj group). The dose of administration was 10^8^ CFU per day per mouse.

**Table 5:** Alpha diversity metrics.

	Control	MS	MS+Lj
Chao 1	274.26 ± 38.46 ^a^	255.71 ± 1.02 ^a^	255.30 ± 8.50 ^a^
Shannon	5.39 ± 0.45 ^a^	5.31 ± 0.13 ^a^	5.05 ± 0.03 ^a^
Observed OTUs (Species)	298.49 ± 23.60 ^a^	266.44 ± 2.76 ^b^	279.02 ± 15.89 ^ab^
PD whole tree	20.87 ± 1.90 ^a^	19.28 ± 0.28 ^a^	19.64 ± 1.08 ^a^

Chao1, Shannon’s diversity, and phylogenetic diversity (PD whole tree) of the fecal contents of the mice fed for 14 weeks with normal diet (Control group), HFD+WB (MS group), and HFD+WB supplemented with *L. johnsonii* CRL1231 (MS+Lj group). Data are expressed as mean ± SE (standard error), *n* = 4 per group. Values with different superscripts within the same row are significantly different (*p* < 0.05).

**Table 6:** Microbial analysis of fecal samples from different mice groups by quantitative PCR (qPCR) at week 14 of feeding.

		Bacterial Counts (log cells/g Fecal Sample)			
Bacterial Groups	Control	MS	*p*-Values ^a^	MS+Lj	*p*-Values ^b^
Total bacteria	10.73	11.39	0.095	10.57	0.093
*Bifidobacterium*	6.71	5.40	0.028 *	6.58	0.033 *
*Bacteroides*	7.40	7.70	0.698	7.60	0.137
*Enterobacteriaceae*	5.44	7.08	0.039 *	7.15	0.148
*Lactobacillus*	7.65	8.29	0.030 *	9.21	0.723

Control: mice receiving normal diet; MS (Metabolic syndrome): mice receiving HFD+WB diet; MS+Lj: mice receiving HFD+WB diet and *L. johnsonii* CRL1231. Data are expressed as the media of Log cells of each bacterial group per gram of stool. ^a^ Differences in values between the Control and the MS groups. ^b^ Differences in values between the MS and the MS+Lj groups. * Significant differences were established at *p* < 0.05 and were indicated with asterisks.

## Data Availability

Sequencing data were deposited in NCBI BioProject—Accession: PRJNA1087445 ID: 1087445. The original contributions to this study are included in the article. For more information, please contact the authors.

## Supplementary Materials

The following supporting information can be downloaded at: https://www.mdpi.com/article/10.3390/metabo15070466/s1, Figure S1: Study design for co-administering *L. johnsonii* CRL1231 and wheat bran to mice with metabolic syndrome. Figure S2: Chromatograms obtained by HPLC-MS after injection of a sample from the MS+Lj group confirm the presence of different metabolites derived from ferulic acid. Table S1: Nutritional composition of diets. Table S2: Oligonucleotide primers used in this study.

## Abbreviations

The following abbreviations are used in this manuscript:

AC	Atherogenic Coefficient
AIP	Atherogenic Index of Plasma
ALT	Alanine Aminotransferase
AST	Aspartate Aminotransferase
AUC	Areas Under the Curve
BA	Benzoic Acid
BWG	Body Weight Gain
CRI	Castelli Risk Index
CVD	Cardiovascular Disease
DHF	Dihydroferulic Acid
DHPPA	3,4-Dihydroxyphenylpropionic acid
FA	Ferulic Acid
FE	Feruloyl Esterase
FER	Food Efficiency Ratio
GPx	Glutathione Peroxidase
GR	Glutathione Reductase
HFD	High Fat Diet
HFD+WB	High-Fat Diet Supplemented with Wheat Bran
HOMA-IR	Homeostasis Model Assessment of Basal Insulin Resistance
HPPA	3-Hydroxyphenylpropionic Acid
IFE	Intestinal Feruloyl Esterase
IM	Intestinal Microbiota
*Lj* CRL1231	*Lactobacillus Johnsonii* CRL1231
MS	Metabolic Syndrome
NAFLD	Non-Alcoholic Fatty Liver Disease
OGTT	Oral Glucose Tolerance Test
OSTT	Oral Sucrose Tolerance Test
SCFAs	Short Chain Fatty Acids
T2D	Type 2 Diabetes
TBARS	Thiobarbituric Acid Reactive Substances
WB	Wheat Bran

## References

[1] Tilg H., Moschen A.R., Ahima R.S. (2023). Gut Microbiome, Obesity, and Metabolic Syndrome. Metabolic Syndrome: A Comprehensive Textbook.

[2] Zimmet P., Alberti K.G.M.M., Stern N., Bilu C., El-Osta A., Einat H., Kronfeld-Schor N. (2019). The Circadian Syndrome: Is the Metabolic Syndrome and Much More. J. Intern. Med..

[3] Tenorio-Jiménez C., Martínez-Ramírez M.J., Gil Á., Gómez-Llorente C. (2020). Effects of Probiotics on Metabolic Syndrome: A Systematic Review of Randomized Clinical Trials. Nutrients.

[4] Wang H.H., Lee D.K., Liu M., Portincasa P., Wang D.Q.-H. (2020). Novel Insights into the Pathogenesis and Management of the Metabolic Syndrome. Pediatr. Gastroenterol. Hepatol. Nutr..

[5] Marventano S., Salomone F., Godos J., Pluchinotta F., Del Rio D., Mistretta A., Grosso G. (2016). Coffee and Tea Consumption in Relation with Non-Alcoholic Fatty Liver and Metabolic Syndrome: A Systematic Review and Meta-Analysis of Observational Studies. Clin. Nutr..

[6] Schwingshackl L., Bogensberger B., Benčič A., Knüppel S., Boeing H., Hoffmann G. (2018). Effects of Oils and Solid Fats on Blood Lipids: A Systematic Review and Network Meta-Analysis. J. Lipid Res..

[7] Yang B., Zheng F., Stanton C., Ross R.P., Zhao J., Zhang H., Chen W. (2021). *Lactobacillus reuteri* FYNLJ109L1 Attenuating Metabolic Syndrome in Mice via Gut Microbiota Modulation and Alleviating Inflammation. Foods.

[8] Zheng F., Wang Z., Stanton C., Ross R.P., Zhao J., Zhang H., Yang B., Chen W. (2021). *Lactobacillus rhamnosus* FJSYC4-1 and *Lactobacillus reuteri* FGSZY33L6 Alleviate Metabolic Syndrome via Gut Microbiota Regulation. Food Funct..

[9] Julibert A., Bibiloni M.d.M., Tur J.A. (2019). Dietary Fat Intake and Metabolic Syndrome in Adults: A Systematic Review. Nutr. Metab. Cardiovasc. Dis..

[10] Zhang F., Qiu L., Xu X., Liu Z., Zhan H., Tao X., Shah N.P., Wei H. (2017). Beneficial Effects of Probiotic Cholesterol-Lowering Strain of *Enterococcus faecium* WEFA23 from Infants on Diet-Induced Metabolic Syndrome in Rats. J. Dairy Sci..

[11] Zheng J., Wittouck S., Salvetti E., Franz C.M.A.P., Harris H.M.B., Mattarelli P., O’Toole P.W., Pot B., Vandamme P., Walter J. (2020). A Taxonomic Note on the Genus Lactobacillus: Description of 23 Novel Genera, Emended Description of the Genus *Lactobacillus* Beijerinck 1901, and Union of Lactobacillaceae and Leuconostocaceae. Int. J. Syst. Evol. Microbiol..

[12] Yang G., Hong E., Oh S., Kim E. (2020). Non-Viable *Lactobacillus johnsonii* JNU3402 Protects against Diet-Induced Obesity. Foods.

[13] Russo M., Marquez A., Abeijón-Mukdsi M.C., Santacruz A., López-Malo A., Gauffin-Cano P., Medina R. (2019). Microencapsulated Feruloyl Esterase-Producing Lactobacilli Ameliorate Lipid Profile and Glycaemia in High Fat Diet-Induced Obese Mice. Benef. Microbes.

[14] Gao J., Gu X., Zhang M., Zu X., Shen F., Hou X., Hao E., Bai G. (2022). Ferulic Acid Targets ACSL1 to Ameliorate Lipid Metabolic Disorders in Db/Db Mice. J. Funct. Foods.

[15] Li D., Rui Y., Guo S., Luan F., Liu R., Zeng N. (2021). Ferulic Acid: A Review of Its Pharmacology, Pharmacokinetics and Derivatives. Life Sci..

[16] Zhao J., Gao J., Li H. (2020). Ferulic Acid Confers Protection on Islet Î^2^ Cells and Placental Tissues of Rats with Gestational Diabetes Mellitus. Cell Mol. Biol..

[17] Li L., Shewry P.R., Ward J.L. (2008). Phenolic Acids in Wheat Varieties in the health grain Diversity Screen. J. Agric. Food Chem..

[18] Turner A.L., Michaelson L.V., Shewry P.R., Lovegrove A., Spencer J.P.E. (2021). Increased Bioavailability of Phenolic Acids and Enhanced Vascular Function Following Intake of Feruloyl Esterase-Processed High Fibre Bread: A Randomized, Controlled, Single Blind, Crossover Human Intervention Trial. Clin. Nutr..

[19] Kasprzak-Drozd K., Oniszczuk T., Stasiak M., Oniszczuk A. (2021). Beneficial Effects of Phenolic Compounds on Gut Microbiota and Metabolic Syndrome. Int. J. Mol. Sci..

[20] Li X., Wang N., Yin B., Fang D., Zhao J., Zhang H., Wang G., Chen W. (2016). *Lactobacillus plantarum* X1 with α-Glucosidase Inhibitory Activity Ameliorates Type 2 Diabetes in Mice. RSC Adv..

[21] Russo M., Marquez A., Herrera H., Abeijon-Mukdsi C., Saavedra L., Hebert E., Gauffin-Cano P., Medina R. (2020). Oral Administration of *Lactobacillus fermentum* CRL1446 Improves Biomarkers of Metabolic Syndrome in Mice Fed a High-Fat Diet Supplemented with Wheat Bran. Food Funct..

[22] Caporaso J.G., Kuczynski J., Stombaugh J., Bittinger K., Bushman F.D., Costello E.K., Fierer N., Peña A.G., Goodrich J.K., Gordon J.I. (2010). QIIME Allows Analysis of High-Throughput Community Sequencing Data. Nat. Methods.

[23] Caporaso J.G., Lauber C.L., Walters W.A., Berg-Lyons D., Huntley J., Fierer N., Owens S.M., Betley J., Fraser L., Bauer M. (2012). Ultra-High-Throughput Microbial Community Analysis on the Illumina HiSeq and MiSeq Platforms. ISME J..

[24] Bokulich N.A., Subramanian S., Faith J.J., Gevers D., Gordon J.I., Knight R., Mills D.A., Caporaso J.G. (2013). Quality-Filtering Vastly Improves Diversity Estimates from Illumina Amplicon Sequencing. Nat. Methods.

[25] Gauffin Cano P., Santacruz A., Trejo F.M., Sanz Y. (2013). Bifidobacterium CECT 7765 Improves Metabolic and Immunological Alterations Associated with Obesity in High-Fat Diet Fed Mice. Obesity.

[26] Deng Y., Yang Q., Hao C., Wang H.H., Ma T., Chen X., Ngai F.-W., Xie Y.J. (2025). Combined Lifestyle Factors and Metabolic Syndrome Risk: A Systematic Review and Meta-Analysis. Int. J. Obes..

[27] Engin A., Engin A.B., Engin A. (2017). The Definition and Prevalence of Obesity and Metabolic Syndrome. Obesity and Lipotoxicity.

[28] Croci S., D’Apolito L.I., Gasperi V., Catani M.V., Savini I. (2021). Dietary Strategies for Management of Metabolic Syndrome: Role of Gut Microbiota Metabolites. Nutrients.

[29] Torres S., Fabersani E., Marquez A., Gauffin-Cano P. (2019). Adipose Tissue Inflammation and Metabolic Syndrome. The Proactive Role of Probiotics. Eur. J. Nutr..

[30] Lee C.S., Park M.H., Kim S.H. (2022). Selection and Characterization of Probiotic Bacteria Exhibiting Antiadipogenic Potential in 3T3-L1 Preadipocytes. Probiotics Antimicro. Prot..

[31] Zhang Y., Mu T., Yang Y., Zhang J., Ren F., Wu Z. (2021). *Lactobacillus johnsonii* Attenuates *Citrobacter rodentium*–Induced Colitis by Regulating Inflammatory Responses and Endoplasmic Reticulum Stress in Mice. J. Nutr..

[32] Han S., Zheng H., Han F., Zhang X., Zhang G., Ma S., Liu K., Qin W., Wu G. (2022). *Lactobacillus johnsonii* 6084 Alleviated Sepsis-Induced Organ Injury by Modulating Gut Microbiota. Food Sci. Nutr..

[33] Fändriks L. (2017). Roles of the Gut in the Metabolic Syndrome: An Overview. J. Intern. Med..

[34] Hu B., Ye C., Leung E.L.-H., Zhu L., Hu H., Zhang Z., Zheng J., Liu H. (2020). *Bletilla striata* Oligosaccharides Improve Metabolic Syndrome through Modulation of Gut Microbiota and Intestinal Metabolites in High Fat Diet-Fed Mice. Pharmacol. Res..

[35] Xin J., Zeng D., Wang H., Ni X., Yi D., Pan K., Jing B. (2014). Preventing Non-Alcoholic Fatty Liver Disease through *Lactobacillus johnsonii* BS15 by Attenuating Inflammation and Mitochondrial Injury and Improving Gut Environment in Obese Mice. Appl. Microbiol. Biotechnol..

[36] Dong L., Qin C., Li Y., Wu Z., Liu L. (2022). Oat Phenolic Compounds Regulate Metabolic Syndrome in High Fat Diet-Fed Mice via Gut Microbiota. Food Biosci..

[37] Wang W., Pan Y., Zhou H., Wang L., Chen X., Song G., Liu J., Li A. (2018). Ferulic Acid Suppresses Obesity and Obesity-Related Metabolic Syndromes in High Fat Diet-Induced Obese C57BL/6J Mice. Food Agric. Immunol..

[38] Mahmoud A.M., Hussein O.E., Hozayen W.G., Bin-Jumah M., Abd El-Twab S.M. (2020). Ferulic Acid Prevents Oxidative Stress, Inflammation, and Liver Injury via Upregulation of Nrf2/HO-1 Signaling in Methotrexate-Induced Rats. Environ. Sci. Pollut. Res..

[39] Teixeira L.D., Torrez Lamberti M.F., DeBose-Scarlett E., Bahadiroglu E., Garrett T.J., Gardner C.L., Meyer J.L., Lorca G.L., Gonzalez C.F. (2021). *Lactobacillus johnsonii* N6.2 and Blueberry Phytophenols Affect Lipidome and Gut Microbiota Composition of Rats Under High-Fat Diet. Front. Nutr..

[40] Qing X., Zeng D., Wang H., Ni X., Liu L., Lai J., Khalique A., Pan K., Jing B. (2017). Preventing Subclinical Necrotic Enteritis through *Lactobacillus johnsonii* BS15 by Ameliorating Lipid Metabolism and Intestinal Microflora in Broiler Chickens. AMB Expr..

[41] Bhardwaj S., Bhattacharjee J., Bhatnagar M.K., Tyagi S., Delhi N. (2013). Atherogenic index of plasma, castelli risk index and atherogenic coefficient-new parameters in assessing cardiovascular risk. Int. J. Pharm. Biol. Sci..

[42] Narasimhan A., Chinnaiyan M., Karundevi B. (2015). Ferulic Acid Exerts Its Antidiabetic Effect by Modulating Insulin-Signalling Molecules in the Liver of High-Fat Diet and Fructose-Induced Type-2 Diabetic Adult Male Rat. Appl. Physiol. Nutr. Metab..

[43] Koh W.Y., Utra U., Ahmad R., Rather I.A., Park Y.-H. (2018). Evaluation of Probiotic Potential and Anti-Hyperglycemic Properties of a Novel *Lactobacillus* Strain Isolated from Water Kefir Grains. Food Sci. Biotechnol..

[44] Serra-Barcellona C., Habib N.C., Honoré S.M., Sánchez S.S., Genta S.B. (2017). Enhydrin Regulates Postprandial Hyperglycemia in Diabetic Rats by Inhibition of α-Glucosidase Activity. Plant Foods Hum. Nutr..

[45] Zhang Z., Yang P., Zhao J. (2022). Ferulic Acid Mediates Prebiotic Responses of Cereal-Derived Arabinoxylans on Host Health. Anim. Nutr..

[46] Bento-Silva A., Koistinen V.M., Mena P., Bronze M.R., Hanhineva K., Sahlstrøm S., Kitrytė V., Moco S., Aura A.-M. (2020). Factors Affecting Intake, Metabolism and Health Benefits of Phenolic Acids: Do We Understand Individual Variability?. Eur. J. Nutr..

[47] Braune A., Bunzel M., Yonekura R., Blaut M. (2009). Conversion of Dehydrodiferulic Acids by Human Intestinal Microbiota. J. Agric. Food Chem..

[48] Duncan S.H., Russell W.R., Quartieri A., Rossi M., Parkhill J., Walker A.W., Flint H.J. (2016). Wheat Bran Promotes Enrichment within the Human Colonic Microbiota of Butyrate-Producing Bacteria That Release Ferulic Acid. Environ. Microbiol..

[49] Wang G., Zhu G., Chen C., Zheng Y., Ma F., Zhao J., Lee Y.-K., Zhang H., Chen W. (2021). *Lactobacillus* Strains Derived from Human Gut Ameliorate Metabolic Disorders via Modulation of Gut Microbiota Composition and Short-Chain Fatty Acids Metabolism. Benef. Microbes.

[50] Sanna S., van Zuydam N.R., Mahajan A., Kurilshikov A., Vich Vila A., Võsa U., Mujagic Z., Masclee A.A.M., Jonkers D.M.A.E., Oosting M. (2019). Causal Relationships among the Gut Microbiome, Short-Chain Fatty Acids and Metabolic Diseases. Nat. Genet..

[51] Tian B., Geng Y., Wang P., Cai M., Neng J., Hu J., Xia D., Cao W., Yang K., Sun P. (2022). Ferulic Acid Improves Intestinal Barrier Function through Altering Gut Microbiota Composition in High-Fat Diet-Induced Mice. Eur. J. Nutr..

[52] González Hernández M.A., Canfora E.E., Jocken J.W.E., Blaak E.E. (2019). The Short-Chain Fatty Acid Acetate in Body Weight Control and Insulin Sensitivity. Nutrients.

[53] Vrieze A., Van Nood E., Holleman F., Salojärvi J., Kootte R.S., Bartelsman J.F.W.M., Dallinga–Thie G.M., Ackermans M.T., Serlie M.J., Oozeer R. (2012). Transfer of Intestinal Microbiota From Lean Donors Increases Insulin Sensitivity in Individuals With Metabolic Syndrome. Gastroenterology.

[54] Lippert K., Kedenko L., Antonielli L., Kedenko I., Gemeier C., Leitner M., Kautzky-Willer A., Paulweber B., Hackl E. (2017). Gut Microbiota Dysbiosis Associated with Glucose Metabolism Disorders and the Metabolic Syndrome in Older Adults. Benef. Microbes.

[55] Bagarolli R.A., Tobar N., Oliveira A.G., Araújo T.G., Carvalho B.M., Rocha G.Z., Vecina J.F., Calisto K., Guadagnini D., Prada P.O. (2017). Probiotics Modulate Gut Microbiota and Improve Insulin Sensitivity in DIO Mice. J. Nutr. Biochem..

[56] Song Y., Wu M., Tao G., Lu M., Lin J., Huang J. (2020). Feruloylated Oligosaccharides and Ferulic Acid Alter Gut Microbiome to Alleviate Diabetic Syndrome. Food Res. Int..

[57] Yan S., Shi R., Li L., Ma S., Zhang H., Ye J., Wang J., Pan J., Wang Q., Jin X. (2019). Mannan Oligosaccharide Suppresses Lipid Accumulation and Appetite in Western-Diet-Induced Obese Mice Via Reshaping Gut Microbiome and Enhancing Short-Chain Fatty Acids Production. Mol. Nutr. Food Res..

[58] Wang H., Ni X., Qing X., Zeng D., Luo M., Liu L., Li G., Pan K., Jing B. (2017). Live Probiotic *Lactobacillus johnsonii* BS15 Promotes Growth Performance and Lowers Fat Deposition by Improving Lipid Metabolism, Intestinal Development, and Gut Microflora in Broilers. Front. Microbiol..

[59] Li H., Liu F., Lu J., Shi J., Guan J., Yan F., Li B., Huo G. (2020). Probiotic Mixture of *Lactobacillus plantarum* Strains Improves Lipid Metabolism and Gut Microbiota Structure in High Fat Diet-Fed Mice. Front. Microbiol..

[60] Zhu Z., Zhu B., Sun Y., Ai C., Wang L., Wen C., Yang J., Song S., Liu X. (2018). Sulfated Polysaccharide from Sea Cucumber and Its Depolymerized Derivative Prevent Obesity in Association with Modification of Gut Microbiota in High-Fat Diet-Fed Mice. Mol. Nutr. Food Res..

[61] Yamaguchi Y., Adachi K., Sugiyama T., Shimozato A., Ebi M., Ogasawara N., Funaki Y., Goto C., Sasaki M., Kasugai K. (2016). Association of Intestinal Microbiota with Metabolic Markers and Dietary Habits in Patients with Type 2 Diabetes. Digestion.

[62] Gong L., Wang H., Wang T., Liu Y., Wang J., Sun B. (2019). Feruloylated Oligosaccharides Modulate the Gut Microbiota in Vitro via the Combined Actions of Oligosaccharides and Ferulic Acid. J. Funct. Foods.

[63] Chávez-Carbajal A., Nirmalkar K., Pérez-Lizaur A., Hernández-Quiroz F., Ramírez-del-Alto S., García-Mena J., Hernández-Guerrero C. (2019). Gut Microbiota and Predicted Metabolic Pathways in a Sample of Mexican Women Affected by Obesity and Obesity Plus Metabolic Syndrome. Int. J. Mol. Sci..

