# Supplementary material for: Amelioration of Metabolic Syndrome by Co-Administration of Lactobacillus johnsonii CRL1231 and Wheat Bran in Mice via Gut Microbiota and Metabolites Modulation
Source: Metabolites. 2025 Jul 9;15(7):466. doi: 10.3390/metabo15070466 (PMC12299901; doi:10.3390/metabo15070466)
Supplement: Supplementary file 1 [file metabolites-15-00466-s001.zip › metabolites-3705438-supplementary.pdf]

Supplementary Materials:

Supplementary Figure S1: Study design for co-administering *L. johnsonii* CRL1231 and wheat bran to mice with metabolic syndrome.

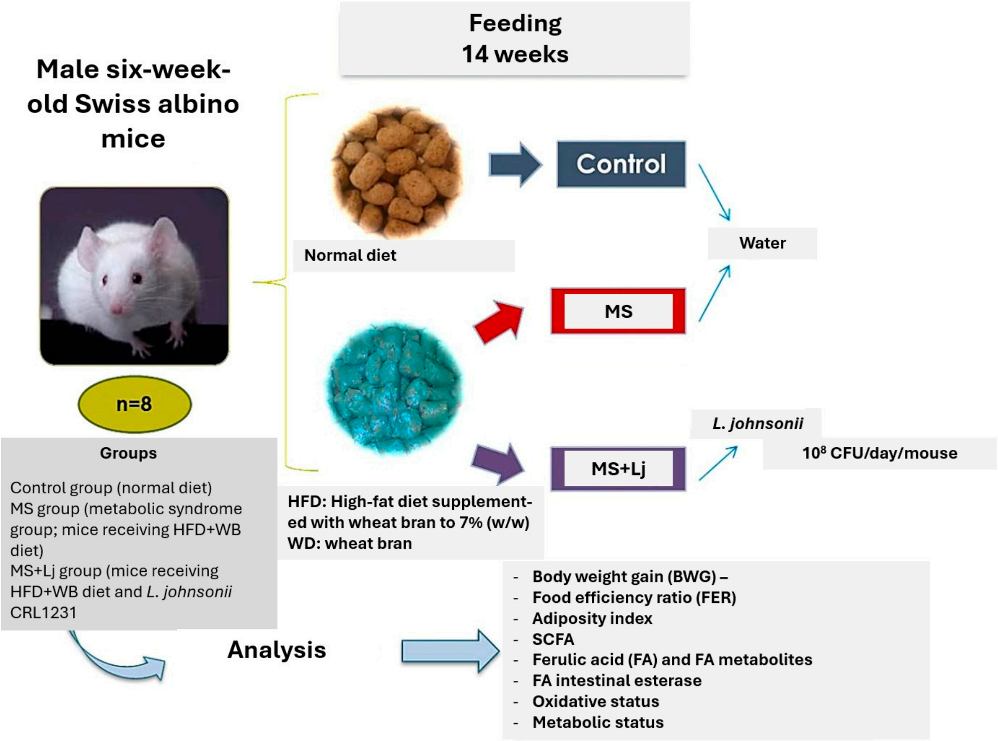

Supplementary Figure S2. Chromatograms obtained by HPLC-MS after injection of a sample from the MS+Lj group confirm the presence of different metabolites derived from ferulic acid.

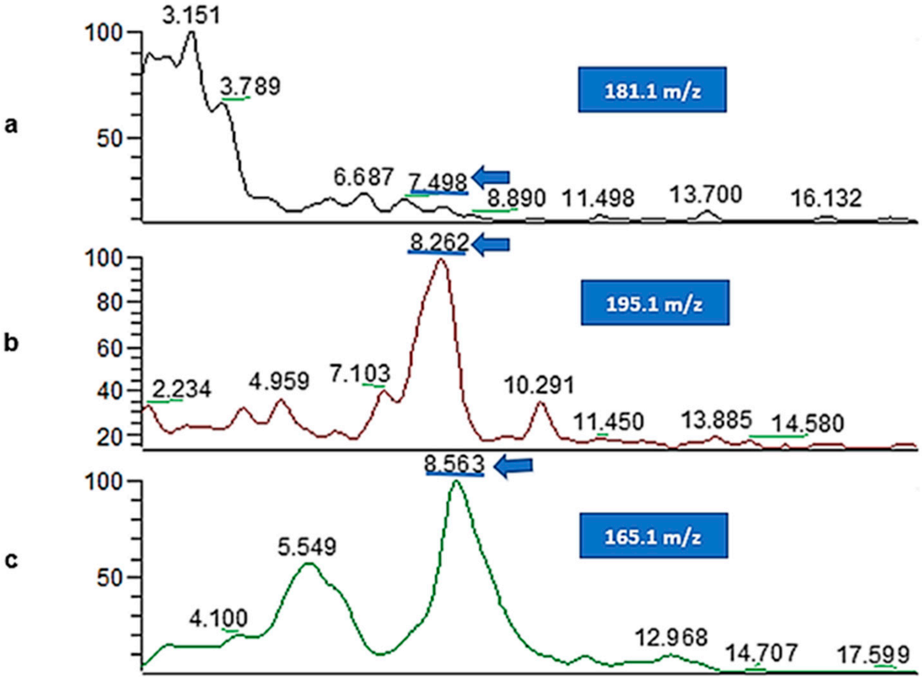

a. Dihydroxyphenylpropionic acid [DHPPA]. **b.** Dihydroferulic acid [DHF]. **c.** Hydroxyphenylpropionic acid [HPPA]. These compounds could be identified considering the mass/charge ratio [m/z] of the ionic species detected (blue boxes) and the retention times [RT] (blue arrows).

Supplementary Table S1. Nutritional composition of diets.

	**Normal diet**	**HFD+WB**
*Moisture (g)	9.89 ± 0.14[a]	5.95 ± 0.13[b]
*Ash (g)	4.57 ± 0.20[a]	3.04 ± 0.19[b]
*Proteins (g)	23.50 ± 0.06[a]	22.15 ± 0.08[b]
*Total Lipids (g)	7.89 ± 0.19[a]	13.20 ± 0.17[b]
*Carbohydrates (g)	38.00 ± 1.55[a]	40.00 ± 0.65[a]
**Kcal/g**	3.10	5.10

*Amounts expressed per 100 g of diet. HFD+WB: High-fat diet supplemented with wheat bran to 7% (w/w). Values with different superscript letters in the same row are significantly different ($P < 0.05$).

Supplementary Table S2. Oligonucleotide primers used in this study.

**Bacterial groups**	**Primers (name)**	**Sequence (5'–3')**	**(pb)**	**Annealing temperature (ºC)**
*Total bacteria	HDA 1, HDA 2	TGGCTCAGGACGAACGCTGGCGGC<br>CCTACTGCTGCCTCCCGTAGGAGT	200	59
**Bifidobacterium*	BiFid F, BiFid R	CTCCTGGAAACGGGTGG<br>GGTGTTCTTCCCGATATCTACA	550	55
**Bacteroides*	Bfra F, Bfra R	ATA GCC TTT CGA AAG RAA GAT<br>CCA GTA TCA ACT GCA ATT TTA	287	55
**Enterobacteriaceae*	Entero 1, Entero 2	CATTGACGTTACCCGCAGAAGAAGC<br>CTCTACGAGACTCAAGCTTGC	195	63
**Lactobacillus*	Lac 1, Lac 2	AGCAGTAGGGAATCTTCCA<br>ATTYCACCGCTACACATG	340	61

*References: Gauffin Cano, MP, Santacruz A, Trejo FM, Sanz, Y (2013) Bifidobacterium CECT 7765 improves metabolic and immunological dysfunction associated with obesity in high-fat diet fed mice. Obes 21: 2310-2321. https://doi.org/10.1002/oby.20330.
